# Immunomodulators for immunocompromised patients hospitalized for COVID-19: a meta-analysis of randomized controlled trials

**DOI:** 10.1016/j.eclinm.2024.102472

**Published:** 2024-02-09

**Authors:** Ilias I. Siempos, Andre C. Kalil, Drifa Belhadi, Viviane Cordeiro Veiga, Alexandre Biasi Cavalcanti, Westyn Branch-Elliman, Eleni Papoutsi, Konstantinos Gkirgkiris, Nikoleta A. Xixi, Anastasia Kotanidou, Olivier Hermine, Raphaël Porcher, Xavier Mariette, CORIMUNO-19 Collaborative Group, DisCoVeRy Study Group, ACTT-2 Study Group, ACTT-3 Study Group, Olivier Hermine, Xavier Mariette, Philippe Ravaud, Serge Bureau, Maxime Dougados, Matthieu Resche-Rigon, Pierre-Louis Tharaux, Annick Tibi, Olivier Hermine, Elie Azoulay, Serge Bureau, Jacques Cadranel, Maxime Dougados, Joseph Emmerich, Muriel Fartoukh, Bertrand Guidet, Marc Humbert, Karine Lacombe, Matthieu Mahevas, Xavier Mariette, Frédéric Pene, Raphaël Porcher, Valerie Pourchet-Martinez, Philippe Ravaud, Matthieu Resche-Rigon, Frédéric Schlemmer, Pierre-Louis Tharaux, Annick Tibi, Yazdan Yazdanpanah, Philippe Ravaud, Raphaël Porcher, Gabriel Baron, Elodie Perrodeau, Serge Bureau, Damien Vanhoye, Cécile Kedzia, Lauren Demerville, Anne Gysembergh-Houal, Alexandre Bourgoin, Matthieu Resche-Rigon, Nabil Raked, Lakhdar Mameri, Claire Montlahuc, Lucie Biard, St.phanie Alary, Samir Hamiria, Thinhinane Bariz, Hala Semri, Dhiaa Meriem Hai, Moustafa Benafla, Mohamed Belloul, Pernelle Vauboin, Saskia Flamand, Claire Pacheco, Anouk Walter-Petrich, Emilia Stan, Souad Benarab, Corine Nyanou, Maxime Dougados, Annick Tibi, Robin Charreteur, Céline Dupre, Kévin Cardet, Blandine Lehmann, Kamyl Baghli, Yazdan Yazdanpanah, Claire Madelaine, Eric D'Ortenzio, Oriane Puéchal, Caroline Semaille, Xavier Mariette, Laurent Savale, Anatole Harrois, Samy Figueiredo, Jacques Duranteau, Nadia Anguel, Arthur Pavot, Xavier Monnet, Christian Richard, Jean-Louis Teboul, Philippe Durand, Pierre Tissieres, Mitja Jevnikar, Marc Humbert, David Montani, Stephan Pavy, Gaétane Nocturne, Samuel Bitoun, Nicolas Noel, Olivier Lambotte, Lelia Escaut, Stephane Jauréguiberry, Elodie Baudry, Christiane Verny, Edouard Lefevre, Mohamad Zaidan, Domitille Molinari, Gaël Leprun, Alain Fourreau, Laurent Cylly, Lamiae Grimaldi, Myriam Virlouvet, Ramdane Meftali, Soléne Fabre, Marion Licois, Asmaa Mamoune, Yacine Boudali, Clotilde Le Tiec, Céline Verstuyft, Anne-Marie Roques, Sophie Georgin-Lavialle, Jacques Cadranel, Patricia Senet, Gilles Pialoux, Angele Soria, Antoine Parrot, Helene François, Nathalie Rozensztajn, Emmanuelle Blin, Pascaline Choinier, Juliette Camuset, Jean-Simon Rech, Antony Canellas, Camille Rolland-Debord, Nadege Lemarié, Nicolas Belaube, Marine Nadal, Martin Siguier, Camille Petit-Hoang, Julie Chas, Elodie Drouet, Matthieu Lemoine, Audrey Phibel, Lucie Aunay, Eliane Bertrand, Sylviane Ravato, Marie Vayssettes, Anne Adda, Celine Wilpotte, Pélagie Thibaut, Julie Fillon, Isabelle Debrix, Soraya Fellahi, Jean-Philippe Bastard, Guillaume Lefévre, Jacques-Eric Gottenberg, Yves Hansmann, Frédéric Blanc, Sophie Ohlmann-Caillard, Vincent Castelain, Emmanuel Chatelus, Eva Chatron, Olivier Collange, François Danion, Frédéric De Blay, Pierre Diemunsch, Sophie Diemunsch, Renaud Felten, Bernard Goichot, Valentin Greigert, Aurelien Guffroy, Bob Heger, Charlotte Kaeuffer, Loic Kassegne, Anne Sophie Korganow, Pierrick Le Borgne, Nicolas Lefebvre, Paul-Michel Mertes, Eric Noll, Mathieu Oberlin, Vincent Poindron, Julien Pottecher, Yvon Ruch, François Weill, Nicolas Meyer, Emmanuel Andres, Eric Demonsant, Hakim Tayebi, Gabriel Nisand, Stéphane Brin, Cédric Sublon, Guillaume Becker, Anne Hutt, Tristan Martin, Sophie Bayer, Catherine Metzger, Arsene Mekinian, Karine Lacombe, Bertrand Guidet, Noémie Abisror, Amir Adedjouma, Diane Bollens, Marion Bonneton, Nathalie Bourcicaux, Anne Bourrier, Maria Chauchard Thibault Chiarabiani, Doroth.e Chopin, Jonathan Cohen, Ines Devred, Bruno Donadille, Olivier Fain, Geoffrey Hariri, Vincent Jachiet, Patrick Ingliz, Marc Garnier, Marc Gatfosse, Etienne Ghrenassia, Delphine Gobert, Bertrand Guidet, Jessica Krause le Garrec, Cecilia Landman, Jean Remy Lavillegrand, Benedicte Lefebvre, Thibault Mahevas, Sandie Mazerand, Jean Luc Meynard, Marjolaine Morgand, Zineb Ouaz.ne, Jerome Pacanowski, S.bastien Riviere, Philippe Seksik, Harry Sokol, Heithem Soliman, Nadia Valin, Thomas Urbina, Chloé McAvoy, Maria Pereira Miranda, Gladys Aratus, Laurence Berard, Tabassome Simon, Anne Daguenel Nguyen, Elise Girault, Cl.mentine Mayala-Kanda, Marie Antignac, Céline Leplay, Gladys Aratus, Laurence Berard, Tabassome Simon, Jean-Benoit Arlet, Jean-Luc Diehl, Florence Bellenfant, Anne Blanchard, Alexandre Buffet, Bernard Cholley, Antoine Fayol, Edouard Flamarion, Anne Godier, Thomas Gorget, Sophie-Rym Hamada, Caroline Hauw-Berlemont, Jean-Sébastien Hulot, David Lebeaux, Marine Livrozet, Adrien Michon, Arthur Neuschwander, Marie-Aude Pennet, Benjamin Planquette, Brigitte Ranque, Olivier Sanchez, Geoffroy Volle, Sandrine Briois, Mathias Cornic, Virginie Elisee, Jesuthasan Denis, Juliette Djadi-Prat, Pauline Jouany, Ramon Junquera, Mickael Henriques, Amina Kebir, Isabelle Lehir, Jeanne Meunier, Florence Patin, Val.rie Paquet, Anne Tréhan, Véronique Vigna, Brigitte Sabatier, Damien Bergerot, Charléne Jouve, Camille Knosp, Olivia Lenoir, Nassim Mahtal, Léa Resmini, Xavier Lescure, Jade Ghosn, Antoine Bachelard, Anne Rachline, Valentina Isernia, Bao-chau, Phung, Dorothée Vallois, Aurelie Sautereau, Catherine Neukrich, Antoine Dossier, Raphaël Borie, Bruno Crestani, Gregory Ducrocq, Philippe Gabriel Steg, Philippe Dieude, Thomas Papo, Estelle Marcault, Marhaba Chaudhry, Charléne Da Silveira, Annabelle Metois, Ismahan Mahenni, Meriam Meziani, Cyndie Nilusmas, Sylvie Le Gac, Awa Ndiaye, Fran.oise Louni, Malikhone Chansombat, Zelie Julia, Solaya Chalal, Lynda Chalal, Laura Kramer, Jeniffer Le Grand, Kafif Ouifiya, Valentine Piquard, Sarah Tubiana, Yann Nguyen, Vasco Honsel, Emmanuel Weiss, Anais Codorniu, Virginie Zarrouk, Victoire de Lastours, Matthieu Uzzan, Naura Gamany, Agathe Claveirole, Alexandre Navid, Tiffanie Fouque, Yonathan Cohen, Maya Lupo, Constance Gilles, Roza Rahli, Zeina Louis, David Boutboul, Lionel Galicier, Elie Azoulay, Lionel Galicier, Yaël Amara, Gabrielle Archer, Elie Azoulay, Amira Benattia, Anne Bergeron, Louise Bondeelle, Nathalie de Castro, Melissa Clément, Michaël Darmon, Blandine Denis, Clairelyne Dupin, Elsa Feredj, Delphine Feyeux, Adrien Joseph, Etienne Lenglin, Pierre Le Guen, Geoffroy Liégeon, Gwenaël Lorillon, Asma Mabrouki, Eric Mariotte, Grégoire Martin de Frémont, Adrien Mirouse, Jean-Michel Molina, Régis Peffault de Latour, Eric Oksenhendler, Julien Saussereau, Abdellatif Tazi, Jean-Jacques Tudesq, Lara Zafrani, Isabelle Brindele, Emmanuelle Bugnet, Karine Celli Lebras, Julien Chabert, Lamia Djaghout, Catherine Fauvaux, Anne Lise Jegu, Ewa Kozakiewicz, Martine Meunier, Marie-Thérèse Tremorin, Claire Davoine, Isabelle Madelaine, Sophie Caillat-Zucman, Constance Delaugerre, Florence Morin, Damien Sène, Ruxandra Burlacu, Benjamin Chousterman, Bruno Mégarbanne, Pascal Richette, Jean-Pierre Riveline, Aline Frazier, Eric Vicaut, Laure Berton, Tassadit Hadjam, Miguel Alejandro Vazquez-Ibarra, Clément Jourdaine, Olivia Tran, Véronique Jouis, Aude Jacob, Julie Smati, Stéphane Renaud, Claire Pernin, Lydia Suarez, Luca Semerano, Sébastien Abad, Ruben B. nainous, Nicolas Bonnet, Celine Comparon, Yves Cohen, Hugues Cordel, Robin Dhote, Nathalie Dournon, Boris Duchemann, Nathan Ebstein, Thomas Gille, Benedicte Giroux-Leprieur, Jeanne Goupil de Bouille, Hilario Nunes, Johanna Oziel, Dominique Roulot, Lucile Sese, ClaireTantet, Yurdagul Uzunhan, Coralie Bloch-Queyrat, Vincent Levy, Fadhila Messani, Mohammed Rahaoui, Myléne Petit, Sabrina Brahmi, Vanessa Rathoin, Marthe Rigal, Nathalie Costedoat-Chalumeau, Liem Binh Luong, Frédéric Pene, Zakaria Ait Hamou, Sarah Benghanem, Philippe Blanche, Nicolas Carlier, Benjamin Chaigne, Remy Gauzit, Hassan Joumaa, Mathieu Jozwiak, Marie Lachétre, Hélène Lafoeste, Odie Launay, Paul Legendre, Jonathan Marey, Caroline Morbieu, Lola-Jade Palmieri, Tali-Anne Szwebel, Hendy Abdoul, Alexandra Bruneau, Audrey Beclin-Clabaux, Charly Larrieu, Pierre Montanari, Eric Dufour, Ada Clarke, Catherine Le Bourlout, Nathalie Marin, Nathalie Menage, Samira Saleh-Mghir, Mamadou Salif Cisse, Kahina Cheref, Corinne Guerin, Jérémie Zerbit, Marc Michel, Sébastien Gallien, Etienne Crickx, Benjamin Le Vavasseur, Emmanuelle Kempf, Karim Jaffal, William Vindrios, Julie Oniszczuk, Marc Michel, Matthieu Mahevas, Constance Guillaud, Frédéric Schlemmer, Pascal Lim, Elena Fois, Giovanna Melica, Marie Matignon, Maud Jalabert, Jean-Daniel Lelièvre, David Schmitz, Marion Bourhis, Sylia Belazouz, Laetitia Languille, Caroline Boucle, Nelly Cita, Agnés Didier, Fahem Froura, Katia Ledudal, Thiziri Sadaoui, Alaki Thiemele, Delphine Le Febvre De Bailly, Muriel Carvhalo Verlinde, Julien Mayaux, Patrice Cacoub, David Saadoun, Mathieu Vautier, Héléne Bugaut, Olivier Benveniste, Yves Allenbach, Gaëlle Leroux, Aude Rigolet, Perrine Guillaume-Jugnot, Fanny Domont, Anne Claire Desbois, Chloé Comarmond, Nicolas Champtiaux, Segolene Toquet, Amine Ghembaza, Matheus Vieira, Georgina Maalouf, Goncalo Boleto, Yasmina Ferfar, Jean-Christophe Corvol, C.line Louapre, Sara Sambin, Louise-Laure Mariani, Carine Karachi, Florence Tubach, Candice Estellat, Linda Gimeno, Karine Martin, Aicha Bah, Vixra Keo, Sabrine Ouamri, Yasmine Messaoudi, Nessima Yelles, Pierre Faye, Sebastien Cavelot, Cecile Larcheveque, Laurence Annonay, Jaouad Benhida, Aida Zahrate-Ghoul, Soumeya Hammal, Ridha Belilita, Fanny Charbonnier, Claire Aguilar, Fanny Alby-Laurent, Carole Burger, Clara Campos-Vega, Nathalie Chavarot, Benjamin Fournier, Claire Rouzaud, Damien Vimpére, Caroline Elie, Prissile Bakouboula, Laure Choupeaux, Sophie Granville, Elodie Issorat, Christine Broissand, Marie-Alexandra Alyanakian, Guillaume Geri, Nawal Derridj, Naima Sguiouar, Hakim Meddah, Mourad Djadel, Héléne Chambrin-Lauvray, Jean-Charles Duclos-vallée, Faouzi Saliba, Sophie-Caroline Sacleux, Ilias Kounis, Sonia Tamazirt, Eric Rudant, Jean-Marie Michot, Annabelle Stoclin, Emeline Colomba, Fanny Pommeret, Christophe Willekens, Rosa Da Silva, Valérie Dejean, Yasmina Mekid, Ines Ben-Mabrouk, Florence Netzer, Caroline Pradon, Laurence Drouard, Valérie Camara-Clayette, Alexandre Morel, Gilles Garcia, Abolfazl Mohebbi, Férial Berbour, Mélanie Dehais, Anne-Lise Pouliquen, Alison Klasen, Loren Soyez-Herkert, Jonathan London, Jonathan London, Younes Keroumi, Emmanuelle Guillot, Guillaume Grailles, Younes El amine, Fanny Defrancq, Hanane Fodil, Chaouki Bouras, Dominique Dautel, Nicolas Gambier, Thierno Dieye, Boris Bienvenu, Victor Lancon, Laurence Lecomte, Kristina Beziriganyan, Belkacem Asselate, Laure Allanic, Elena Kiouris, Marie-Héléne Legros, Christine Lemagner, Pascal Martel, Vincent Provitolo, Félix Ackermann, Mathilde Le Marchand, Aurélie Chan Hew Wai, Dimitri Fremont, Elisabeth Coupez, Mireille Adda, Frédéric Duée, Lise Bernard, Antoine Gros, Estelle Henry, Claire Courtin, Anne Pattyn, Pierre-Grégoire Guinot, Marc Bardou, Agnes Maurer, Julie Jambon, Amélie Cransac, Corinne Pernot, Bruno Mourvillier, Eric Marquis, Philippe Benoit, Damien Roux, Coralie Gernez, Cécile Yelnik, Julien Poissy, Mandy Nizard, Fanette Denies, Helene Gros, Jean-Jacques Mourad, Emmanuelle Sacco, Sophie Renet, F. Ader, Y. Yazdanpanah, F. Mentre, N. Peiffer-Smadja, F.X. Lescure, J. Poissy, L. Bouadma, J.F. Timsit, B. Lina, F. Morfin-Sherpa, M. Bouscambert, A. Gaymard, G. Peytavin, L. Abel, J. Guedj, C. Andrejak, C. Burdet, C. Laouenan, D. Belhadi, A. Dupont, T. Alfaiate, B. Basli, A. Chair, S. Laribi, J. Level, M. Schneider, M.C. Tellier, A. Dechanet, D. Costagliola, B. Terrier, M. Ohana, S. Couffin-Cadiergues, H. Esperou, C. Delmas, J. Saillard, C. Fougerou, L. Moinot, L. Wittkop, C. Cagnot, S. Le Mestre, D. Lebrasseur-Longuet, V. Petrov-Sanchez, A. Diallo, N. Mercier, V. Icard, B. Leveau, S. Tubiana, B. Hamze, A. Gelley, M. Noret, E. D’Ortenzio, O. Puechal, C. Semaille, T. Welte, J.A. Paiva, M. Halanova, M.P. Kieny, E. Balssa, C. Birkle, S. Gibowski, E. Landry, A. Le Goff, L. Moachon, C. Moins, L. Wadouachi, C. Paul, A. Levier, D. Bougon, F. Djossou, L. Epelboin, J. Dellamonica, C.H. Marquette, C. Robert, S. Gibot, E. Senneville, V. Jean-Michel, Y. Zerbib, C. Chirouze, A. Boyer, C. Cazanave, D. Gruson, D. Malvy, P. Andreu, J.P. Quenot, N. Terzi, K. Faure, C. Chabartier, V. Le Moing, K. Klouche, T. Ferry, F, Valour, B. Gaborit, E. Canet, P. Le Turnier, D. Boutoille, F. Bani-Sadr, F. Benezit, M. Revest, C. Cameli, A. Caro, MJ Ngo Um Tegue, Y. Le Tulzo, B. Laviolle, F. Laine, G. Thiery, F. Meziani, Y. Hansmann, W. Oulehri, C. Tacquard, F. Vardon-Bounes, B. Riu-Poulenc, M. Murris-Espin, L. Bernard, D. Garot, O. Hinschberger, M. Martinot, C. Bruel, B. Pilmis, O. Bouchaud, P. Loubet, C. Roger, X. Monnet, S. Figueiredo, V. Godard, J.P. Mira, M. Lachatre, S. Kerneis, J. Aboab, N. Sayre, F. Crockett, D. Lebeaux, A. Buffet, J.L. Diehl, A. Fayol, J.S. Hulot, M. Livrozet, A Mekontso- Dessap, C. Ficko, F. Stefan, J. Le Pavec, J. Mayaux, H. Ait-Oufella, J.M. Molina, G. Pialoux, M. Fartoukh, J. Textoris, M. Brossard, A. Essat, E. Netzer, Y. Riault, M. Ghislain, L. Beniguel, M. Genin, L. Gouichiche, L. Moinot, C. Betard, L. Wittkop, L. Belkhir, A. Altdorfer, V Fraipont Centro, S. Braz, JM Ferreira Ribeiro, J.A. Paiva, R Roncon Alburqueque, M. Berna, M. Alexandre, B. Lamprecht, A. Egle, R. Greil, R. Greil, M. Joannidis, Thomas F. Patterson, Philip O. Ponce, Barbara S. Taylor, Jan E. Patterson, Jason E. Bowling, Heta Javeri, Andre C. Kalil, LuAnn Larson, Angela Hewlett, Aneesh K. Mehta, Nadine G. Rouphael, Youssef Saklawi, Nicholas Scanlon, Jessica J. Traenkner, Ronald P. Trible, Emmanuel B. Walter, Noel Ivey, Thomas L. Holland, Guillermo M. Ruiz-Palacios, Alfredo Ponce de León, Sandra Rajme, Lanny Hsieh, Alpesh N. Amin, Miki Watanabe, Helen S. Lee, Susan Kline, Joanne Billings, Brooke Noren, Hyun Kim, Tyler D. Bold, Victor Tapson, Jonathan Grein, Fayyaz Sutterwala, Nicole Iovine, Lars K. Beattie, Rebecca Murray Wakeman, Matthew Shaw, Mamta K. Jain, Satish Mocherla, Jessica Meisner, Amneris Luque, Daniel A. Sweeney, Constance A. Benson, Farhana Ali, Robert L. Atmar, Hana M. El Sahly, Jennifer Whitaker, Ann R. Falsey, Angela R. Branche, Cheryl Rozario, Justino Regalado Pineda, José Arturo Martinez-Orozco, David Chien Lye, Sean WX. Ong, Po Ying Chia, Barnaby E. Young, Uriel Sandkovsky, Mezgebe Berhe, Clinton Haley, Emma Dishner, Valeria D. Cantos, Colleen F. Kelley, Paulina A. Rebolledo Esteinou, Sheetal Kandiah, Sarah B. Doernberg, Pierre-Cedric B. Crouch, Hannah Jang, Anne F. Luetkemeyer, Jay Dwyer, Stuart H. Cohen, George R. Thompson, Hien H. Nguyen, Robert W. Finberg, Jennifer P. Wang, Juan Perez-Velazquez, Mireya Wessolossky, Patrick E.H. Jackson, Taison D. Bell, Miranda J. West, Babafemi Taiwo, Karen Krueger, Johnny Perez, Triniece Pearson, Catharine I. Paules, Kathleen G. Julian, Danish Ahmad, Alexander G. Hajduczok, Henry Arguinchona, Christa Arguinchona, Nathaniel Erdmann, Paul Goepfert, Neera Ahuja, Maria G. Frank, David Wyles, Heather Young, Myoung-don Oh, Wan Beom Park, Chang Kyung Kang, Vincent Marconi, Abeer Moanna, Sushma Cribbs, Telisha Harrison, Eu Suk Kim, Jongtak Jung, Kyoung-Ho Song, Hong Bin Kim, Seow Yen Tan, Humaira Shafi, Jaime Chien, Raymond KC. Fong, Daniel D. Murray, Jens Lundgren, Henrik Nielsen, Tomas Jensen, Barry S. Zingman, Robert Grossberg, Paul F. Riska, Otto O. Yang, Jenny Ahn, Rubi Arias, Rekha R. Rapaka, Naomi Hauser, James D. Campbell, William R. Short, Pablo Tebas, Jillian T. Baron, Susan L.F. McLellan, Lucas S. Blanton, Justin B. Seashore, C. Buddy Creech, Todd W. Rice, Shannon Walker, Isaac P. Thomsen, Diego Lopez de Castilla, Jason W. Van Winkle, Francis X. Riedo, Surinder Kaur Pada, Alvin DY. Wang, Li Lin, Michelle Harkins, Gregory Mertz, Nestor Sosa, Louis Yi Ann Chai, Paul Anantharajah Tambyah, Sai Meng Tham, Sophia Archuleta, Gabriel Yan, David A. Lindholm, Ana Elizabeth Markelz, Katrin Mende, Richard Mularski, Elizabeth Hohmann, Mariam Torres-Soto, Nikolaus Jilg, Ryan C. Maves, Gregory C. Utz, Sarah L. George, Daniel F. Hoft, James D. Brien, Roger Paredes, Lourdes Mateu, Cora Loste, Princy Kumar, Sarah Thornton, Sharmila Mohanraj, Noreen A. Hynes, Lauren M. Sauer, Christopher J. Colombo, Christina Schofield, Rhonda E. Colombo, Susan E. Chambers, Richard M. Novak, Andrea Wendrow, Samir K. Gupta, Tida Lee, Tahaniyat Lalani, Mark Holodniy, Aarthi Chary, Nikhil Huprikar, Anuradha Ganesan, Norio Ohmagari, Ayako Mikami, D. Ashley Price, Christopher J.A. Duncan, Kerry Dierberg, Henry J. Neumann, Stephanie N. Taylor, Alisha Lacour, Najy Masri, Edwin Swiatlo, Kyle Widmer, James D. Neaton, Mary Bessesen, David S. Stephens, Timothy H. Burgess, Timothy M. Uyeki, Robert Walker, G. Lynn Marks, Anu Osinusi, Huyen Cao, Anabela Cardoso, Stephanie de Bono, Douglas E. Schlichting, Kevin K. Chung, Jennifer L. Ferreira, Michelle Green, Mat Makowski, Michael R. Wierzbicki, Tom M. Conrad, Jill Ann El-Khorazaty, Heather Hill, Tyler Bonnett, Nikki Gettinger, Theresa Engel, Teri Lewis, Jing Wang, John H. Beigel, Kay M. Tomashek, Varduhi Ghazaryan, Tatiana Beresnev, Seema Nayak, Lori E. Dodd, Walla Dempsey, Effie Nomicos, Marina Lee, Rhonda Pikaart-Tautges, Mohamed Elsafy, Robert Jurao, Hyung Koo, Michael Proschan, Tammy Yokum, Janice Arega, Ruth Florese, Jocelyn D. Voell, Richard Davey, Andre C. Kalil, LuAnn Larson, Angela Hewlett, Thomas F. Patterson, Philip O. Ponce, Jan E. Patterson, Barbara S. Taylor, Jason E. Bowling, Ruth C. Serrano, Aneesh K. Mehta, Jessica J. Traenkner, Nadine G. Rouphael, Zanthia Wiley, Varun K. Phadke, Nathaniel Erdmann, Paul A. Goepfert, Carlos A. Gomez, Theresa A. Sofarelli, Laura Certain, Hannah N. Imlay, Cameron R. Wolfe, Emily R. Ko, John J. Engemann, Emmanuel B. Walter, Mamta K. Jain, Satish Mocherla, Jessica Meisner, Guillermo M. Ruiz-Palacios, Alfredo Ponce de León, Sandra Rajme, Susan Kline, Joanne Billings, Hyun Kim, Justino Regalado Pineda, José Arturo Martinez-Orozco, Nora Bautista Felix, Claire R. Wan, Sammy T. Elmor, Laurel R. Bristow, Michelle S. Harkins, Gregory Mertz, Nestor Sosa, Patrick E.H. Jackson, Taison D. Bell, Miranda J. West, Nicole M. Iovine, Marie-Carmelle Elie-Turenne, Victor F. Tapson, Jonathan Grein, Fayyaz Sutterwala, Myoung-don Oh, Pyoeng Gyun Choe, Chang Kyung Kang, Richard A. Mularski, Catharine I. Paules, Kevin S. Rhie, Rezhan H. Hussein, Dilek Ince, Patricia L. Winokur, Jin Takasaki, Ayako Mikami, Sho Saito, Daniel A. Sweeney, Constance A. Benson, Kimberly McConnell, Uriel Sandkovsky, Mezgebe Berhe, Emma Dishner, David L. Wyles, Maria G. Frank, Ellen Sarcone, Mamta K. Jain, Satish Mocherla, Jessica Meisner, Sarah B. Doernberg, Pierre-Cedric B. Crouch, Hannah Jang, Elizabeth Hohmann, Nikolaus Jilg, Kevin A. Grimes, Katherine Perez, Charles Janak, Hana M. El Sahly, Jennifer A. Whitaker, Valeria D. Cantos, Paulina A. Rebolledo, John Gharbin, Robert Grossberg, Barry S. Zingman, Paul F. Riska, Allison A. Lambert, Henry Arguinchona, Christa Arguinchona, Diego Lopez de Castilla, Jason W. Van Winkle, Diego F. Zea, Eu Suk Kim, Jongtak Jung, Kyoung-Ho Song, Hong Bin Kim, Anne F. Luetkemeyer, Jay Dwyer, Emma Bainbridge, David C. Hostler, Jordanna M. Hostler, Brian T. Shahan, Lanny Hsieh, Alpesh N. Amin, Miki Watanabe, William R. Short, Pablo Tebas, Jillian T. Baron, Neera Ahuja, Evelyn Ling, Minjoung Go, Otto O. Yang, Jenny Ahn, Rubi Arias, Rekha R. Rapaka, Fleesie A. Hubbard, James D. Campbell, Stuart H. Cohen, Melony Chakrabarty, Maryrose Laguio-Vila, Edward E. Walsh, Ann R. Falsey, Stephanie N. Taylor, Najy Masri, Alisha Lacour, Tida Lee, Tahaniyat Lalani, Hana M. El Sahly, Jennifer A. Whitaker, David A. Lindholm, Ana Elizabeth Markelz, Katrin Mende, Angela R. Branche, Christopher J. Colombo, Christina Schofield, Rhonda E. Colombo, Faheem Guirgis, Mark Holodniy, Aarthi Chary, Mary Bessesen, Noreen A. Hynes, Lauren M. Sauer, Vincent C. Marconi, Abeer Moanna, Telisha Harrison, David Chien Lye, Sean WX. Ong, Po Ying Chia, Nikhil Huprikar, Anuradha Ganesan, Christian Madar, Richard M. Novak, Andrea Wendrow, Scott A. Borgetti, Sarah L. George, Daniel F. Hoft, James D. Brien, Susan L.F. McLellan, Corri Levine, Joy Nock, Seow Yen Tan, Humaira Shafi, Jaime Chien, Keith Candiotti, Robert W. Finberg, Jennifer P. Wang, Mireya Wessolossky, Ryan C. Maves, Gregory C. Utz, Susan E. Chambers, Timothy H. Burgess, Julia Rozman, Fernando Dangond, Yann Hyvert, Andrea Seitzinger, Anu Osinusi, Huyen Cao, Kevin K. Chung, Jennifer L. Ferreira, Michelle Green, Mat Makowski, Tom M. Conrad, Kaitlyn Cross, Jill Ann El-Khorazaty, Heather Hill, Stephanie Pettibone, Michael R. Wierzbicki, Tyler Bonnett, Nikki Gettinger, Theresa Engel, Teri Lewis, Jing Wang, John H. Beigel, Kay M. Tomashek, Varduhi Ghazaryan, Tatiana Beresnev, Seema U. Nayak, Lori E. Dodd, Walla Dempsey, Gregory A. Deye, Effie Nomicos, Rhonda Pikaart-Tautges, Mohamed Elsafy, Robert Jurao, Hyung Koo, Michael Proschan, Richard Davey, Tammy Yokum, Janice Arega, Ruth Florese

**Affiliations:** a First Department of Critical Care Medicine and Pulmonary Services, Evangelismos Hospital, National and Kapodistrian University of Athens Medical School, Athens, Greece; b Department of Medicine, Division of Pulmonary and Critical Care Medicine, Weill Cornell Medicine, New York, NY, USA; c Division of Infectious Diseases, Department of Internal Medicine, University of Nebraska Medical Center, Omaha, NE, USA; d Département d'Épidémiologie, Biostatistiques et Recherche Clinique, Assistance Publique Hôpitaux de Paris, Hôpital Bichat, Paris, France; e Université Paris Cité, Inserm, IAME, Paris F-75018, France; f BP-A Beneficência Portuguesa de São Paulo, São Paulo, Brazil; g Brazilian Research in Intensive Care Network (BRICNet), São Paulo, Brazil; h HCor Research Institute, São Paulo, Brazil; i Department of Medicine, VA Boston Healthcare System, Boston, MA, USA; j Harvard Medical School, Boston, MA, USA; k Département d'hématologie, Hôpital Necker, Assistance Publique Hôpitaux de Paris, Université de Paris, Institut Imagine, INSERM U1183, Paris, France; l Centre de Recherche Épidémiologie et Statistique Sorbonne Paris Cité (CRESS-UMR1153), Inserm / Université Paris, Centre d'épidémiologie Clinique, Hôpital Hôtel-Dieu, France; m Département de Rhumatologie, Hôpital Bicêtre, Assistance Publique Hôpitaux de Paris, Université Paris Saclay, INSERM UMR 1184, Le Kremlin Bicêtre, France

**Keywords:** Acute respiratory distress syndrome, Acute hypoxemic respiratory failure, Pneumonia, Critically ill, Cancer

## Abstract

**Background:**

Although immunomodulators have established benefit against the new coronavirus disease (COVID-19) in general, it is uncertain whether such agents improve outcomes without increasing the risk of secondary infections in the specific subgroup of previously immunocompromised patients. We assessed the effect of immunomodulators on outcomes of immunocompromised patients hospitalized for COVID-19.

**Methods:**

The protocol was prospectively registered with PROSPERO (CRD42022335397). MEDLINE, Cochrane Central Register of Controlled Trials and references of relevant articles were searched up to 01-06-2022. Authors of potentially eligible randomized controlled trials were contacted to provide data on immunocompromised patients randomized to immunomodulators vs control (i.e., placebo or standard-of-care).

**Findings:**

Eleven randomized controlled trials involving 397 immunocompromised patients hospitalized for COVID-19 were included. Ten trials had low risk of bias. There was no difference between immunocompromised patients randomized to immunomodulators vs control regarding mortality [30/182 (16.5%) vs 41/215 (19.1%); RR 0.93, 95% CI 0.61–1.41; p = 0.74], secondary infections (RR 1.00, 95% CI 0.64–1.58; p = 0.99) and change in World Health Organization ordinal scale from baseline to day 15 (weighed mean difference 0.27, 95% CI -0.09–0.63; p = 0.15). In subgroup analyses including only patients with hematologic malignancy, only trials with low risk of bias, only trials administering IL-6 inhibitors, or only trials administering immunosuppressants, there was no difference between comparators regarding mortality.

**Interpretation:**

Immunomodulators, compared to control, were not associated with harmful or beneficial outcomes, including mortality, secondary infections, and change in ordinal scale, when administered to immunocompromised patients hospitalized for COVID-19.

**Funding:**

10.13039/501100013209Hellenic Foundation for Research and Innovation.

**Research in context**

**Evidence before this study**

Relevant guidelines acknowledge the uncertainty regarding the effect of immunomodulators against the new coronavirus disease (COVID-19) in the specific subgroup of previously immunocompromised patients.

Three investigators systematically searched MEDLINE and the Cochrane Central Register of Controlled Trials to identify randomized controlled trials, which tested immunomodulators vs control (i.e., placebo or standard-of-care) in patients hospitalized for COVID-19, enrolled immunocompromised patients and reported data on all-cause mortality. Databases and reference lists of the initially retrieved articles were searched up to June 1st, 2022, without language restrictions.

Out of the 55 initially identified randomized controlled trials, none specifically reported data on the effect of immunomodulators on outcomes of immunocompromised patients with COVID-19.

**Added value of this study**

After contacting authors of the initially identified randomized controlled trials, we clarified that 11 trials (reported in 10 articles), involving 397 immunocompromised patients hospitalized for COVID-19 (182 immunomodulators, 215 control), provided data on clinical outcomes.

We found no statistically significant difference between immunocompromised patients randomized to immunomodulators vs control regarding mortality, secondary infections and change in World Health Organization ordinal scale from baseline to day 15. The main findings persisted in several subgroup analyses.

**Implications of all the available evidence**

The findings of this meta-analysis (albeit based on imprecise estimates due to limited number of events) are compatible with a benefit of the same magnitude as observed for the general population of COVID-19 patients. Therefore, these findings may support the guidelines, which recommend immunomodulators for immunocompromised patients similar to the general population.

That being said, given that immunocompromised patients remain potentially at risk for developing severe COVID-19 even at this stage of the pandemic since they respond worst to vaccination, randomized controlled trials specifically testing the effect of immunomodulators on outcomes of previously immunocompromised patients hospitalized for COVID-19 are needed.

## Introduction

Although several immunomodulators have established benefit against the new coronavirus disease (COVID-19) in general,^1^, ^2^, ^3^ it is uncertain whether such agents improve outcomes of the specific subgroup of previously immunocompromised patients. The latter subgroup of patients remains important because they may experience blunted immunological responses to vaccines.^4^

While acknowledging that additional immunomodulation in immunocompromised patients might increase the risk of secondary infections, relevant guidelines from the United States Centers for Disease Control and Prevention (CDC) state that “for most hospitalized patients with COVID-19 who are immunocompromised, the Panel recommends using immunomodulatory therapies at the doses and durations that are recommended for the general population”.^5^ However, this recommendation was based on limited evidence, because immunocompromised patients were either excluded from or poorly represented in major clinical trials.^5^ Given that even major individual clinical trials may lack power to identify a true effect of immunomodulators in immunocompromised patients, a meta-analytic approach may provide a distinct opportunity to better define this effect.

Accordingly, a meta-analysis was conducted to assess the effect of immunomodulators on clinical outcomes (i.e., mortality, secondary infections, and clinical response) of immunocompromised patients hospitalized for COVID-19.

## Methods

The meta-analysis was reported in accordance with the Preferred Reporting Items for Systematic Reviews and Meta-Analyses (PRISMA) statement.

### Identification of trials

The protocol of the meta-analysis was prospectively registered with PROSPERO (CRD42022335397). Three investigators (NAX, KG and EP) systematically searched MEDLINE and the Cochrane Central Register of Controlled Trials using the following search phrase: (COVID-19 OR “Coronavirus disease 19″ OR SARS-CoV-2 OR “severe acute respiratory syndrome coronavirus 2″) AND (dexamethasone OR corticosteroid∗ OR steroid∗ OR tocilizumab OR anakinra OR baricitinib OR interferon OR immunosuppressant). Databases and reference lists of the initially retrieved articles were searched up to June 1st, 2022, without language restrictions.

Randomized controlled trials, which tested immunomodulators vs control (i.e., placebo or standard-of-care) in patients hospitalized for COVID-19, enrolled immunocompromised patients and reported data on all-cause mortality, were considered eligible. Observational studies, case reports, editorials and reviews; trials involving outpatients; trials excluding immunocompromised patients and trials testing inhaled agents were excluded.

As immunomodulators for COVID-19 were considered the following: systemic steroids, interleukin (IL)-1 inhibitors, IL-6 inhibitors, Janus kinase inhibitors, granulocyte-macrophage colony-stimulating factor inhibitors, LIGHT inhibitors, interferon beta-1a and tumour necrosis factor inhibitors.

Immunocompromised patients were defined by the presence of any of the following medical conditions prior to the diagnosis of COVID-19: hematologic malignancy (lymphoid or myeloid hemopathy, or haematopoietic stem cell transplantation); active solid malignancy; solid organ transplantation; auto-immune disorder under immunomodulators; pre-hospital immunomodulatory treatment; human immunodeficiency virus/acquired immunodeficiency syndrome not on highly active antiretroviral therapy and primary immune deficiency.

### Development of meta-analysis

Corresponding authors and/or funding agencies (such as the United States National Institute of Allergy and Infectious Diseases) of potentially eligible randomized controlled trials were contacted. After confirming that their trials indeed enrolled more than one immunocompromised patient, authors were invited to participate in the meta-analysis and to provide relevant data.

The following data from each trial were retrieved: acronym (or first author); year of publication; intervention; total number of enrolled patients; number of immunocompromised patients among enrolled; number of patients with hematologic cancer among enrolled; median age; sex and outcomes.

The primary outcome of the meta-analysis was all-cause 28-day mortality. Secondary outcomes were secondary infections and change in World Health Organization (WHO) ordinal scale from baseline to day 15 (Supplementary eTable S1).

Two authors (EP and KG) independently assessed the risk of bias for all-cause 28-day mortality (i.e., the primary outcome) for the retrieved randomized controlled trials, using “Cochrane Risk of Bias tool for randomized trials (RoB 2)”.^6^ The RoB 2 tool consisted of the following five domains: 1) the randomization process, 2) the deviations from the intended interventions, 3) the missing outcome data, 4) the measurement of the outcome, and 5) the selection of the reported results. Accordingly, the trials were categorized as having “low risk of bias” or “some concerns” or “high risk of bias”.

Two authors (EP and KG) independently utilized the Grading of Recommendations Assessment, Development, and Evaluation (GRADE) to assess the certainty of the evidence regarding the effect of administration of immunomodulators on mortality of immunocompromised patients hospitalized for COVID-19. Any disagreements regarding the risk of bias or certainty of the evidence were discussed with the corresponding author (IIS).

### Statistical analysis

Data synthesis was conducted using Review Manager 5.4 (Cochrane Collaboration). Pooled dichotomous effect measures were expressed as risk ratio (RR) with 95% confidence intervals (CI) and pooled continuous effect measures as weighed mean difference with 95% CI. Change in ordinal scale from baseline to day 15 was calculated by subtracting the ordinal scale of day 15 from the baseline ordinal scale. Continuous values presented as medians were transformed to means, as instructed by the Cochrane Handbook version 6.3, 2022. Statistical heterogeneity was assessed with I^2^, corresponding to the percentage of the variability in effect measures due to heterogeneity between studies. An inverse variance random-effects model was utilized. A p value < 0.05 denoted statistical significance.

### Role of the funding source

The funding source had no role in the design and conduct of the study; collection, management, analysis, and interpretation of the data; preparation, review, or approval of the manuscript; and decision to submit the manuscript for publication.

## Results

**Fig. 1:**
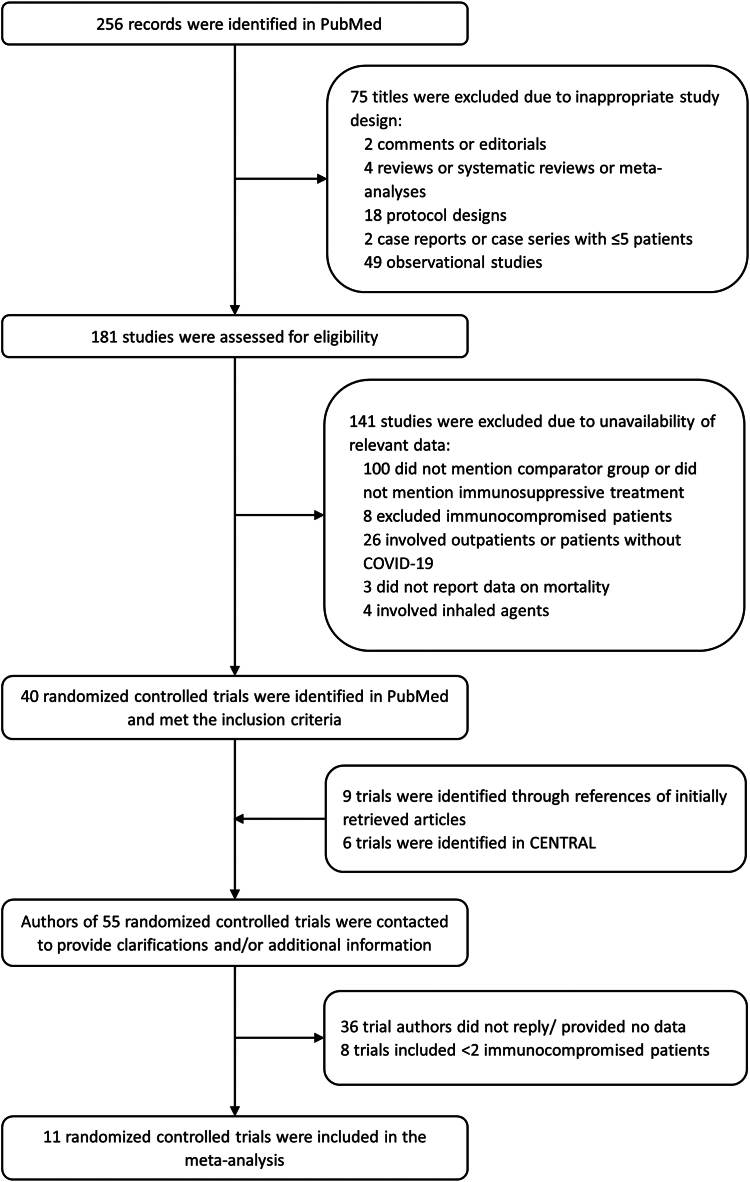
Study flow diagram.

**Fig. 2:**
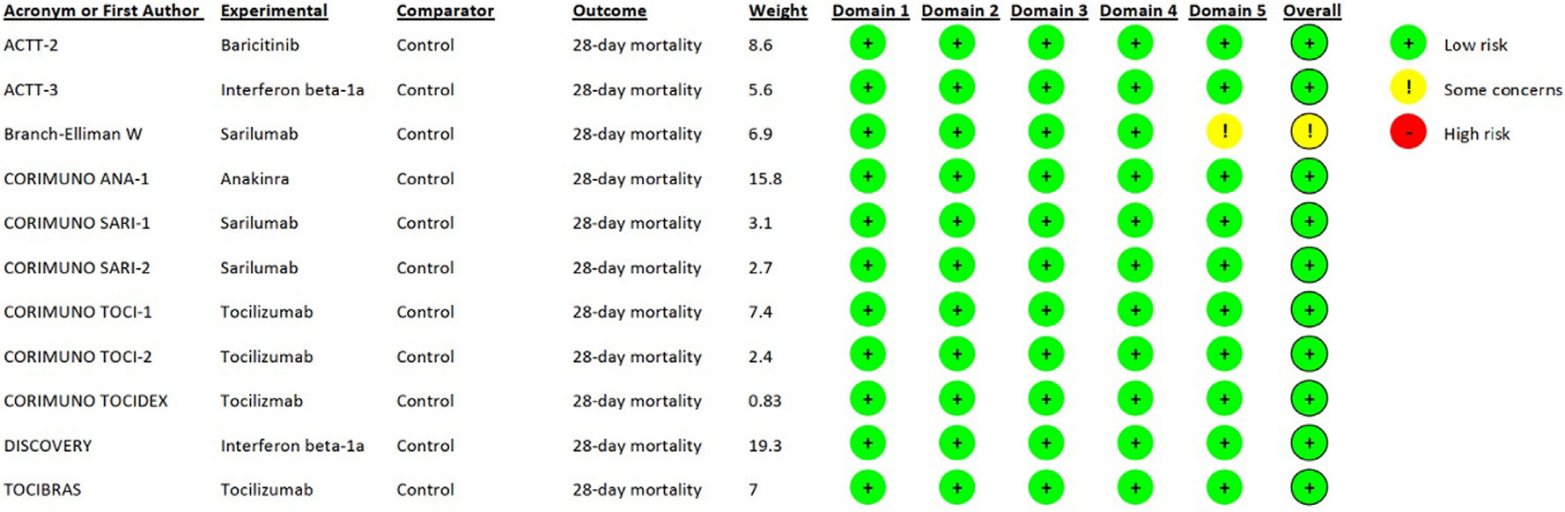
Risk of bias assessment of included randomized controlled trials. Risk of bias for the primary outcome of the meta-analysis (namely, all-cause 28-day mortality) was assessed using the “Cochrane Risk of Bias tool for randomized trials (RoB 2)”. The tool consisted of five domains: 1) the randomization process, 2) the deviations from the intended interventions, 3) the missing outcome data, 4) the measurement of the outcome, and 5) the selection of the reported results. Each domain had up to seven questions. The green circles represent “low risk of bias”, the yellow circles represent “some concerns”, and the red “high risk of bias”.

Fig. 1 shows the flow diagram for study selection. Out of the 55 initially identified randomized controlled trials, none specifically reported data on the effect of immunomodulators on outcomes of immunocompromised patients with COVID-19. After contacting authors of the trials, it was clarified that eight trials included zero or one immunocompromised patients. Finally, 11 randomized controlled trials (reported in 10 articles), involving 397 immunocompromised patients hospitalized for COVID-19 (182 immunomodulators, 215 control), were incorporated in our meta-analysis.^7^, ^8^, ^9^, ^10^, ^11^, ^12^, ^13^, ^14^, ^15^, ^16^ Table 1 and Table 2 summarize baseline characteristics and outcomes of included patients, respectively. Fig. 2 summarizes risk of bias assessment of the included trials. Ten trials had low risk of bias,^7^^,^^8^^,^^10^, ^11^, ^12^, ^13^, ^14^, ^15^, ^16^ while the remaining trial had some concerns regarding the selection of the reported results.^9^

**Table 1:** Characteristics of included randomized controlled trials and immunocompromised patients with COVID-19 randomized to immunomodulators vs control.

Acronym or First Author	Year	Intervention	Total number of patients	Number of immunocompromised patients	Number of patients with hematologic cancer	Age (years)	Female sex	Baseline ordinal scale
ACTT-2^6^	2021	Baricitinib	445 vs 446	33 (7.6) vs 27 (6.3)	NA	62.0 (53.0–67.5) vs 65.5 (56.5–77.5)	11 (33.3) vs 8 (29.6)	5.0 (5.0–6.0) vs 5.0 (5.0–5.0)
ACTT-3^7^	2021	Interferon beta-1a	403 vs 414	60 (15.1) vs 66 (15.9)	NA	67.5 (54.3–74.0) vs 68.0 (56.0–77.5)	33 (55.0) vs 39 (59.1)	5.0 (5.0–5.0) vs 5.0 (5.0–5.0)
Branch-Elliman^8^	2022	Sarilumab	20 vs 30	3 (15.0) vs 1 (3.3)	1 (33.3) vs 0 (0.0)	76.9 (75.8–89.9) vs 75.5	0 (0.0) vs 0 (0.0)	5.0 (5.0–5.0) vs 5.0
CORIMUNO ANA-1^9^	2021	Anakinra	59 vs 55	9 (15.3) vs 9 (16.3)	6 (67.0) vs 2 (22.2)	67.0 (66.1–72.7) vs 69.3 (63.4–82.8)	4 (44.4) vs 4 (44.4)	5.0 (5.0–5.0) vs 5.0 (5.0–5.0)
CORIMUNO SARI-1^10^	2021	Sarilumab	68 vs 76	12 (17.6) vs 9 (11.8)	3 (25.0) vs 5 (55.5)	63.2 (50.2–75.0) vs 68.0 (65.1–72.3)	4 (33.3) vs 3 (33.3)	5.0 (5.0–5.0) vs 5.0 (5.0–5.0)
CORIMUNO SARI-2^11^	2022	Sarilumab	50 vs 41	4 (8.0) vs 4 (9.7)	1 (25.0) vs 1 (25.0)	57.2 (52.1–61.5) vs 56.1 (50.7–64.2)	2 (50.0) vs 1 (25.0)	6.5 (6.0–7.0) vs 6.5 (6.0–7.0)
CORIMUNO TOCI-1^12^	2021	Tocilizumab	63 vs 67	11 (17.5) vs 15 (22.4)	5 (45.5) vs 3 (20.0)	67.2 (63.0–71.9) vs 63.4 (60.4–69.0)	4 (36.4) vs 6 (40.0)	5.0 (5.0–5.0) vs 5.0 (5.0–5.0)
CORIMUNO TOCI-2^11^	2022	Tocilizumab	51 vs 46	7 (13.7) vs 5 (10.9)	3 (42.9) vs 2 (40.0)	61.8 (60.2–65.9) vs 66.8 (65.8–67.4)	3 (42.9) vs 0 (0.0)	7.0 (7.0–7.0) vs 7.0 (6.0–7.0)
CORIMUNO TOCIDEX^13^	2022	Tocilizumab	224 vs 226	18 (8.0) vs 21 (9.3)	5 (27.8) vs 6 (28.6)	67.0 (54.4–73.1) vs 73.2 (57.5–78.0)	5 (27.8) vs 5 (23.8)	5.0 (5.0–5.0) vs 5.0 (5.0–5.0)
DISCOVERY^14^	2021	Interferon beta-1a	147 vs 446	14 (9.6) vs 47 (10.5)	1 (7.2) vs 6 (12.8)	64.0 (53.0–71.0) vs 63.0 (54.0–71.0)	44 (29.9) vs 128 (28.7)	4.0 (4.0–6.0) vs 4.0 (4.0–6.0)
TOCIBRAS^15^	2021	Tocilizumab	65 vs 64	11 (17.2) vs 11 (17.2)	1 (10.0) vs 0 (0.0)	64.8 (55.2–77.2) vs 52.9 (43.4–69.3)	2 (18.2) vs 5 (45.5)	6.0 (5.0–6.0) vs 5.0 (5.0–7.0)

NA, not available/applicable.

Data are expressed as numbers (%) or median (interquartile range).

In ACTT-2 and ACTT-3 trials, data on baseline immunocompromised status were missing for 27 (8 in the immunomodulators vs 19 in the control group) and 8 (5 in the immunomodulators vs 3 in the control group) patients, respectively. In those trials, patients with cancer were considered to be immunocompromised.

**Table 2:** Outcomes of immunocompromised patients with COVID-19 randomized to immunomodulators vs control.

Acronym or First author	Mortality of immunocompromised patients	Mortality of patients with hematologic cancer	Secondary infections	Change in ordinal scale from baseline to day 15
ACTT-2	5 (15.2) vs 3 (11.1)	NA	ΝΑ	3.0 (1.0–4.0) vs 3.0 (0.0–3.0)
ACTT-3	2 (3.3) vs 4 (6.1)	NA	ΝΑ	3.0 (3.0–3.0) vs 3.0 (3.0–3.0)
Branch-Elliman	0 (0.0) vs 0 (0.0)	NA	1 (33.3) vs 1 (100.0)	NA
CORIMUNO ANA-1	3 (33.3) vs 7 (77.8)	2 (33.3) vs 2 (100.0)	1 (11.1) vs 1 (11.1)	0.0 (−2.0 to 3.0) vs −3.0 (−3.0 to −3.0)
CORIMUNO SARI-1	2 (16.7) vs 1 (11.1)	1 (33.3) vs 0 (0)	1 (8.3) vs 1 (11.1)	0.0 (0.0–1.0) vs 0.0 (−1.0 to 0.0)
CORIMUNO SARI-2	1 (25.0) vs 1 (25.0)	1 (100.0) vs 1 (100.0)	0 (0) vs 0 (0)	2.5 (−0.2 to 5.0) vs 1.0 (−0.2 to 2.8)
CORIMUNO TOCI-1	2 (18.2) vs 5 (33.3)	1 (20.0) vs 1 (33.3)	0 (0) vs 3 (20.0)	1.0 (0.0–4.0) vs −2.0 (−2.0 to 4.0)
CORIMUNO TOCI-2	1 (14.3) vs 1 (20.0)	0 (0) vs 1 (50.0)	2 (28.6) vs 2 (40.0)	0.0 (0.0–1.5) vs 0.0 (−1.0 to 1.0)
CORIMUNO TOCIDEX	5 (27.8) vs 7 (33.3)	1 (20.0) vs 1 (16.7)	2 (11.1) vs 5 (23.8)	4.0 (0.0–4.0) vs 0.5 (−2.2 to 4.0)
DISCOVERY	5 (35.8) vs 10 (29.8)	0 (0) vs 3 (50.0)	7 (5%) vs 18 (4%)	1.0 (0.0–2.0) vs 2.0 (0.0–2.0)
TOCIBRAS	4 (36.4) vs 2 (18.2)	NA	5 (45.5) vs 3 (27.3)	−1.0 (−2.0 to 4.0) vs 2.0 (0.0–4.0)

NA, not available/applicable.

Data are expressed as numbers (%) or median (interquartile range).

Change in ordinal scale from baseline to day 15 was calculated by subtracting the ordinal scale of day 15 (or, if not available, day 14) from the baseline ordinal scale.

In TOCIBRAS trial, patients discharged home were considered to have score 1 on the ordinal scale (i.e., “not hospitalized-no limitations on activities”), and those hospitalized without oxygen were considered to have score 3 (i.e., “hospitalized, not requiring supplemental oxygen—no longer requires ongoing medical care”).

**Fig. 3:**
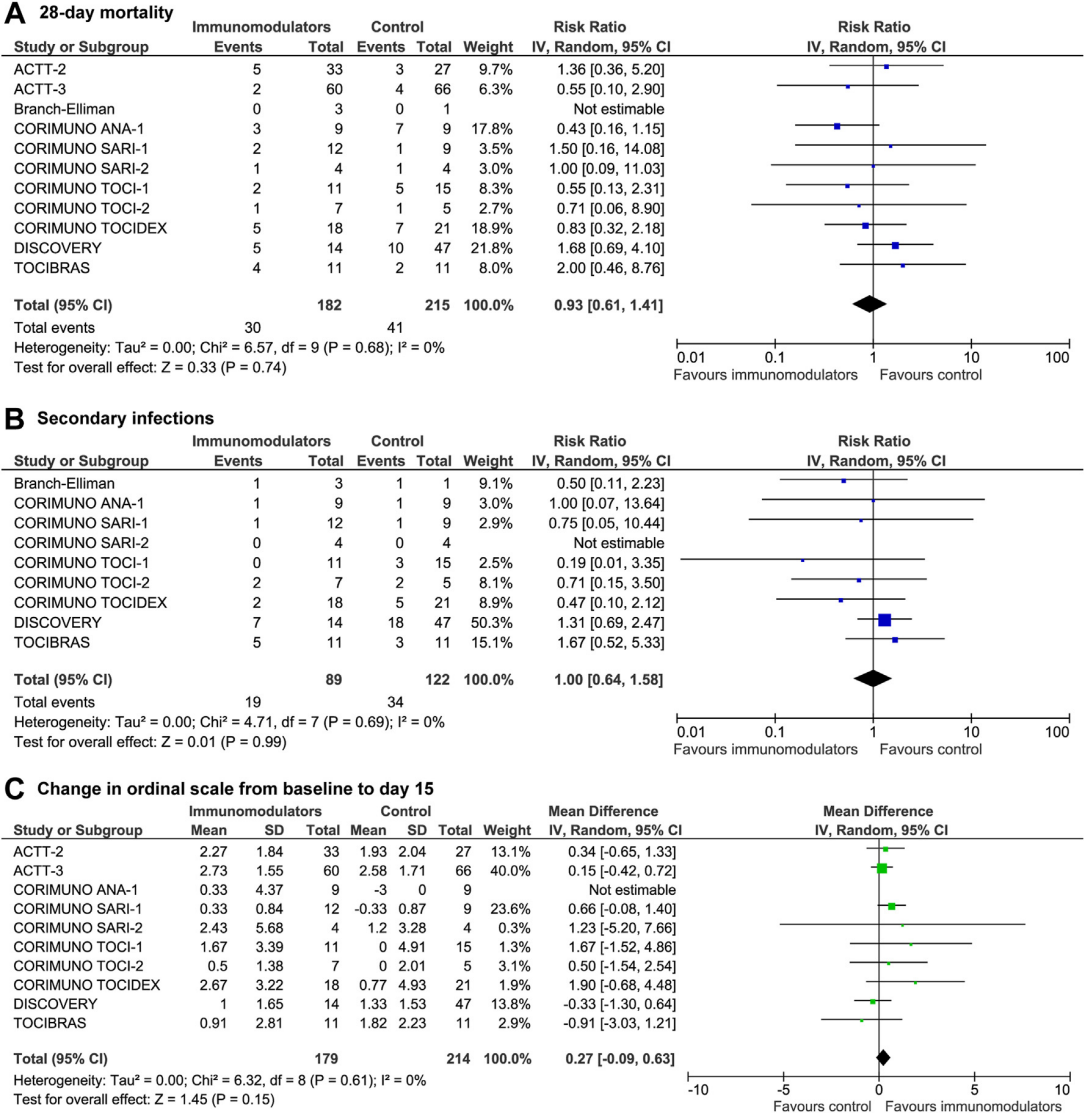
Comparison of immunocompromised patients with COVID-19 randomized to immunomodulators vs control in terms of (A) all-cause 28-day mortality, (B) secondary infections, and (C) change in ordinal scale from baseline to day 15. Pooled risk ratio (RR) and 95% confidence intervals (CI) were calculated using a random-effects model.

All 11 trials provided data on mortality.^7^, ^8^, ^9^, ^10^, ^11^, ^12^, ^13^, ^14^, ^15^, ^16^ No statistical heterogeneity was detected (I^2^ = 0%). There was no difference between immunocompromised patients randomized to immunomodulators vs control regarding mortality [71 deaths; 30/182 (16.5%) vs 41/215 (19.1%); RR 0.93, 95% CI 0.61–1.41, p = 0.74; Fig. 3A]. The certainty of evidence was low (Supplementary eTable S2).

Nine trials provided data on secondary infections.^9^, ^10^, ^11^, ^12^, ^13^, ^14^, ^15^, ^16^ No statistical heterogeneity was detected (I^2^ = 0%). There was no difference between comparators regarding secondary infections (53 events; 21.3% vs 27.9%; RR 1.00, 95% CI 0.64–1.58, p = 0.99; Fig. 3B).

Ten trials provided data on change in ordinal scale from baseline to day 15.^7^^,^^8^^,^^10^, ^11^, ^12^, ^13^, ^14^, ^15^, ^16^ No statistical heterogeneity was detected (I^2^ = 0%). There was no difference between comparators regarding change in ordinal scale from baseline to day 15 (393 patients; weighed mean difference 0.27, 95% CI -0.09–0.63, p = 0.15; Fig. 3C).

**Fig. 4:**
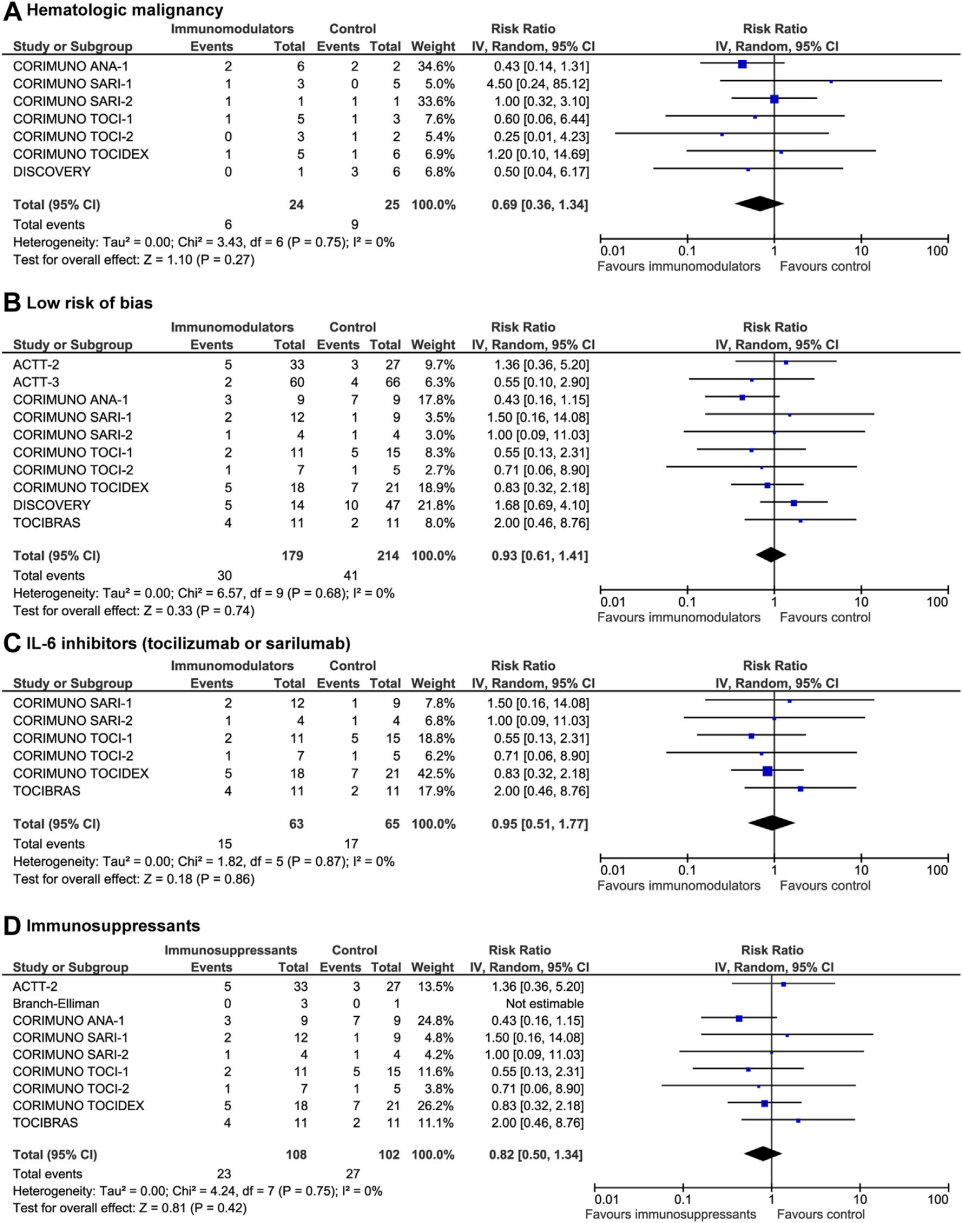
All-cause 28-day mortality of immunocompromised patients with COVID-19 randomized to immunomodulators vs control in the subgroup analyses (A) including only immunocompromised patients with hematologic malignancy, (B) including only trials with low risk of bias, (C) including only trials administering IL-6 inhibitors, and (D) including only trials administering immunosuppressants. Pooled risk ratio (RR) and 95% confidence intervals (CI) were calculated using a random-effects model.

There were no differences in terms of mortality between comparators in the subgroup analyses including only immunocompromised patients with hematologic malignancy (7 trials^10^, ^11^, ^12^, ^13^, ^14^, ^15^; 15 deaths; 25.0% vs 36.0%; RR 0.69, 95% CI 0.36–1.34, p = 0.27; Fig. 4A), including only trials with low risk of bias (10 trials^7^^,^^8^^,^^10^, ^11^, ^12^, ^13^, ^14^, ^15^, ^16^; 71 deaths; 16.8% vs 19.2%; RR 0.93, 95% CI 0.61–1.41, p = 0.74; Fig. 4B), only trials administering IL-6 inhibitors (6 trials^11^, ^12^, ^13^, ^14^^,^^16^; 32 deaths; 23.8% vs 26.2%; RR 0.95, 95% CI 0.51–1.77, p = 0.86; Fig. 4C), or only trials administering immunosuppressants (i.e., after exclusion of trials administering interferon beta-1a which aimed to boost the antiviral response) (9 trials^7^^,^^9^, ^10^, ^11^, ^12^, ^13^, ^14^^,^^16^; 50 deaths; 21.3% vs 26.5%; RR 0.82, 95% CI 0.50–1.34, p = 0.42; Fig. 4D).

Consistently, there were no differences in terms of mortality between comparators in the subgroup analyses including only immunocompromised patients receiving supplemental oxygen (9 trials^7^, ^8^, ^9^, ^10^, ^11^^,^^13^, ^14^, ^15^, ^16^; 47 deaths; 11.5% vs 17.1%; RR 0.73, 95% CI 0.44–1.22, p = 0.23) or including only immunocompromised patients with severe/critical COVID-19 receiving advanced respiratory support (namely, high-flow nasal oxygen, mechanical ventilation or extracorporeal membrane oxygenation) (6 trials^7^^,^^8^^,^^12^^,^^15^^,^^16^; 23 deaths; 39.4% vs 28.6%; RR 1.39, 95% CI 0.71–2.72, p = 0.33).

## Discussion

By incorporating data from 11 randomized controlled trials (10 of them with low risk of bias) involving 397 immunocompromised patients hospitalized for COVID-19, this meta-analysis found no statistically significant difference between patients randomized to immunomodulators vs control in terms of mortality, secondary infections, and change in ordinal scale.

We found that none of the initially retrieved randomized controlled trials specifically reported data on the effect of immunomodulators on outcomes of immunocompromised patients with COVID-19. This finding may justify statements from experts, who emphasized that immunocompromised patients have been neglected in COVID-19 randomized controlled trials and called for action.^17^^,^^18^ Examples of such action may be a preferential inclusion of immunocompromised patients in ongoing platform trials, a focus on safety (such as secondary infections) and the implementation of innovative trial design (such as basket trials), which takes into consideration the variability of immunocompromised conditions.^17^ To generate relevant evidence in a timely manner, the above action should leverage the collective research expertise of health centers who care for such patients.^19^ As an example of such collaborative research effort may serve the present meta-analysis.

Given that immunocompromised patients represented an extreme minority of participants or were completely excluded from trials testing immunomodulators against COVID-19, evidence to inform decisions in such patients is currently derived from observational studies^20^, ^21^, ^22^ or, most commonly, is extrapolated from studies involving populations with physiologic immunological baselines.^18^ In this context, the present meta-analysis might represent a comprehensive attempt to inform decisions regarding the management of immunocompromised patients based on data from randomized controlled trials.

Although the present meta-analysis revealed no significant effect in the group of immunocompromised patients, the estimates were imprecise due to limited number of events and, therefore, solid recommendations cannot be made. That being said, the estimates of the meta-analysis are compatible with a benefit of the same magnitude as observed for the general population of COVID-19 patients. This might mean that enhancing immunosuppression in already immunocompromised patients may not alter the development of specific responses aimed to clear the virus; a reassuring conjecture given that such patients often experience high viral loads and prolonged virologic clearance.^23^ Taken together, the results of the meta-analysis may have a direct implication for clinical practice by supporting the guidelines, which recommend immunomodulators for immunocompromised patients similar to the general population.^5^ While keeping this general guidance into mind, clinicians caring for immunocompromised patients should appreciate that the evidence basis informing the use of immunomodulators in this population is limited and, thus, clinical decision making for how best to manage these patients is complicated and individual risk/benefit analysis may be needed to inform bedside practice.^18^

This meta-analysis has limitations. Firstly, not all authors of individual trials responded to our invitation to participate in the meta-analysis. However, the most likely explanation for non-responsiveness may be that those authors had not included any immunocompromised patients. Secondly, no trials evaluating steroid monotherapy were included in the meta-analysis. Finally, we lacked detailed data on the extent or type of immunosuppression and, therefore, as previously in the literature,^5^^,^^18^^,^^19^ we considered patients with a variety of different immunocompromised conditions as a single risk group. We alert the reader that the study population of immunocompromised patients was heterogenous with potentially differential outcomes. Indeed, in sepsis not related to COVID-19, patients with bone marrow transplantation may have higher mortality,^24^ but patients with solid organ transplantation may have lower mortality,^25^ when compared to non-transplant patients. In COVID-19, evidence suggests that patients with some types of immunosuppression (e.g., associated with ongoing receipt of cytotoxic chemotherapy or receipt of multiple immunosuppressive medications) remain at higher risk of severe disease when compared to patients with other risk profiles.^26^^,^^27^ It is possible that patients with different types of immunosuppression may exhibit differential treatment responses to immunomodulators that were not able to measure in this study. Patients with different types of immunosuppression also have variable antibody response to mRNA vaccination against COVID-19.^28^ However, this heterogeneity of our study population reflects clinical practice and the main findings were consistent among subgroup analyses.

Immunomodulators, compared to control, were not associated with harmful or beneficial outcomes, including mortality, secondary infections, and change in ordinal scale, when administered to immunocompromised patients hospitalized for COVID-19. However, uncertainty due to imprecision indicates that randomized controlled trials more inclusive of immunocompromised patients are needed.

## Contributors

IIS and EP had full access to all of the data in the study and take responsibility for the integrity of the data and the accuracy of the data analysis. IIS, OH, RP and XM conceived of and designed the study. ACK, DB, VCV, ABC, WBE, EP, KG, NAX, AK, OH, RP and XM contributed to acquisition, analysis, or interpretation of data. IIS and EP drafted the manuscript. ACK, DB, VCV, ABC, WBE, KG, NAX, AK, OH, RP and XM contributed to critical revision of the manuscript for important intellectual content. EP performed the statistical analyses. IIS obtained funding and supervised the study.

## Data sharing statement

As this was a meta-analysis of already published randomized controlled trials, no new datasets were produced.

## Declaration of interests

ACK was investigator for the National Institutes of Health Adaptive COVID-19 Treatment Trial. WBE was the site Principal Investigator for a therapeutics study funded by Gilead Sciences (funds to institution) during the past three years. WBE also reports grant funding support from the VA Health Services Research and Development Service (VA HSRD IIR 20-101, 20-076) and from the VA National Artificial Intelligence Institute. OH reports research funds from Roche. The remaining authors (IIS, DB, VCV, ABC, EP, KG, NAX, AK, RP, XM) report no conflicts of interest.

## Appendix ASupplementary data

Supplementary eTables S1 and S2

Supplement- List of Collaborators
