# Supplementary material for: Immunomodulators for immunocompromised patients hospitalized for COVID-19: a meta-analysis of randomized controlled trials
Source: eClinicalMedicine. 2024 Feb 9;69:102472. doi: 10.1016/j.eclinm.2024.102472 (PMC10867612; doi:10.1016/j.eclinm.2024.102472)
Supplement: Supplementary eTables S1 and S2 [file mmc1.docx]

**Supplement**

**Title:** Immunomodulators for immunocompromised patients hospitalized for COVID-19: A meta-analysis of randomized controlled trials

**Authors:** Ilias I. Siempos, Andre C. Kalil, Drifa Belhadi, Viviane Cordeiro Veiga, Alexandre Biasi Cavalcanti, Westyn Branch-Elliman, Eleni Papoutsi, Konstantinos Gkirgkiris, Nikoleta A. Xixi, Anastasia Kotanidou, Olivier Hermine, Raphaël Porcher, Xavier Mariette; CORIMUNO-19 Collaborative Group; DisCoVeRy Study Group; ACTT-2 Study Group; ACTT-3 Study Group

**eTable 1.** **World Health Organization ordinal scale utilized in the meta-analysis**…page 2

**eTable 2. Grading of Recommendations Assessment, Development, and Evaluation (GRADE) assessment**……………………………………………………………………page 3

| **eTable 1. World Health Organization ordinal scale utilized in the meta-analysis** | |
| --- | --- |
| 1 | Not hospitalized, no limitations on activities |
| 2 | Not hospitalized, limitation on activities and/or requiring home oxygen |
| 3 | Hospitalized, not requiring supplemental oxygen – no longer requires ongoing medical care |
| 4 | Hospitalized, not requiring supplemental oxygen – requiring ongoing medical care |
| 5 | Hospitalized, requiring supplemental oxygen |
| 6 | Hospitalized, on high-flow nasal oxygen therapy or non-invasive mechanical ventilation |
| 7 | Hospitalized, on mechanical ventilation or extracorporeal membrane oxygenation |
| 8 | Death |

**eTable 2.** Grading of Recommendations Assessment, Development, and Evaluation (GRADE) assessment.

| **Certainty assessment** | | | | | | | **No. of patients** | | **Effect** | | **Certainty** | **Importance** |
| --- | --- | --- | --- | --- | --- | --- | --- | --- | --- | --- | --- | --- |
| **No. of studies** | **Study design** | **Risk of bias** | **Inconsistency** | **Indirectness** | **Imprecision** | **Other considerations** | **Immunomodulators** | **Control** | **Relative (95% CI)** | **Absolute (95% CI)** |  |  |
| **28-day mortality** | | | | | | | | | | | | |
| 11 | randomized trials | not serious | not serious | not serious | very serious^a^ | none | 30/182 (16.5%) | 41/215 (19.1%) | **RR 0.93** (0.61 to 1.41) | **13 fewer per 1.000** (from 74 fewer to 78 more) | ⨁⨁◯◯ Low | CRITICAL |

**No.:** number; **CI:** confidence interval; **RR:** risk ratio

Imprecision was downgraded by 2 levels because the 95% of the relative risk (RR) was sufficiently wide that the estimate could either include appreciable harm or benefit of the intervention (thresholds: 0.61 and 1.41), and the optimal information size criterion is not met.
