# Supplementary material for: Immunomodulators for immunocompromised patients hospitalized for COVID-19: a meta-analysis of randomized controlled trials
Source: eClinicalMedicine. 2024 Feb 9;69:102472. doi: 10.1016/j.eclinm.2024.102472 (PMC10867612; doi:10.1016/j.eclinm.2024.102472)
Supplement: Supplement- List of Collaborators [file mmc2.docx]

**Supplement- List of Collaborators**

**Title:** Immunomodulators for immunocompromised patients hospitalized for COVID-19: A meta-analysis of randomized controlled trials

**Authors:** Ilias I. Siempos, Andre C. Kalil, Drifa Belhadi, Viviane Cordeiro Veiga, Alexandre Biasi Cavalcanti, Westyn Branch-Elliman, Eleni Papoutsi, Konstantinos Gkirgkiris, Nikoleta A. Xixi, Anastasia Kotanidou, Olivier Hermine, Raphaël Porcher, Xavier Mariette; CORIMUNO-19 Collaborative Group; DisCoVeRy Study Group; ACTT-2 Study Group; ACTT-3 Study Group

1. **CORIMUNO-19 Collaborative Group**……….pages 2-11
2. **DisCoVeRy Study Group**………………………pages 12-14
3. **ACTT-2 Study Group**………………………….pages 15-20
4. **ACTT-3 Study Group**……………………….....pages 21-26

**A. CORIMUNO-19 Collaborative Group**

**Steering Committee**

Olivier Hermine, Xavier Mariette, Philippe Ravaud (chair of the CORIMUNO-19 platform), Serge Bureau, Maxime Dougados, Matthieu Resche-Rigon, Pierre-Louis Tharaux, Annick Tibi

**Scientific Committee**

Olivier Hermine (chair), Elie Azoulay, Serge Bureau, Jacques Cadranel, Maxime Dougados, Joseph Emmerich, Muriel Fartoukh, Bertrand Guidet, Marc Humbert, Karine Lacombe, Matthieu Mahevas, Xavier Mariette, Frédéric Pene, Raphaël Porcher, Valerie Pourchet-Martinez, Philippe Ravaud, Matthieu Resche-Rigon, Frédéric Schlemmer, Pierre-Louis Tharaux, Annick Tibi, Yazdan Yazdanpanah

**Methodology and statistics**

*Methodology:* Philippe Ravaud

*Statistics:* Raphaël Porcher (statistics lead), Gabriel Baron, Elodie Perrodeau (internal independent statistician)

**CORIMUNO-19 Central Coordinating Office: DRCI – AP-HP**

*Responsible for the sponsor:* Serge Bureau

Damien Vanhoye, Cécile Kedzia, Lauren Demerville, Anne Gysembergh-Houal, Alexandre Bourgoin

**CORIMUNO-19 platform trials Coordinating Office:** DRCI – Unités de Recherche Clinique Lariboisiére / Fernand Widal / Saint Louis, AP-HP

*Clinical Trial Unit Lead:* Matthieu Resche-Rigon

*Clinical Trial Unit Manager:* Nabil Raked, Lakhdar Mameri,

*Clinical Trial Unit team:* Claire Montlahuc, Lucie Biard, St.phanie Alary, Samir Hamiria, Thinhinane Bariz, Hala Semri, Dhiaa Meriem Hai, Moustafa Benafla, Mohamed Belloul, Pernelle Vauboin, Saskia Flamand, Claire Pacheco, Anouk Walter-Petrich, Emilia Stan, Souad Benarab, Corine Nyanou

*Trial logistics support:* Maxime Dougados, Université de Paris, Hôpital Cochin, AP-HP

**Drug supply: AGEPS – AP-HP**

*Pharmacy lead:* Annick Tibi

Robin Charreteur, Céline Dupre, Kévin Cardet, Blandine Lehmann, Kamyl Baghli

**REACTing Consortium** (Coordination between AP-HP, Inserm and Universities)

*Chair:* Yazdan Yazdanpanah

Claire Madelaine, Eric D'Ortenzio, Oriane Puéchal, Caroline Semaille

**Local clinical centres of CORIMUNO-19 trials staff** (listed in order of the number of patients randomized per site in the totality of CORIMUNO-19 trials)

**AP-HP, Hôpital Bicêtre, Université Paris-Saclay**

***PI:*** Xavier Mariette, ***Co-Pi:*** Laurent Savale

***Investigators:*** Anatole Harrois, Samy Figueiredo, Jacques Duranteau, Nadia Anguel, Arthur Pavot, Xavier Monnet, Christian Richard, Jean-Louis Teboul, Philippe Durand, Pierre Tissieres, Mitja Jevnikar, Marc Humbert, David Montani, Stephan Pavy, Gaétane Nocturne, Samuel Bitoun, Nicolas Noel, Olivier Lambotte, Lelia Escaut, Stephane Jauréguiberry, Elodie Baudry, Christiane Verny, Edouard Lefevre, Mohamad Zaidan

***Local Clinical Research unit:*** Domitille Molinari, Gaël Leprun, Alain Fourreau, Laurent Cylly, Lamiae Grimaldi

***Local Clinical Research team:*** Myriam Virlouvet, Ramdane Meftali, Soléne Fabre, Marion Licois, Asmaa Mamoune, Yacine Boudali

***Pharmacy:*** Clotilde Le Tiec

***Biological resource centre:*** Céline Verstuyft, Anne-Marie Roques

**AP-HP, Hôpital Tenon, Université Paris-Sorbonne**

***PI:*** Sophie Georgin-Lavialle, ***Co-Pi:*** Jacques Cadranel

***Investigators:*** Patricia Senet, Gilles Pialoux, Angele Soria, Antoine Parrot, Helene François, Nathalie Rozensztajn, Emmanuelle Blin, Pascaline Choinier, Juliette Camuset, Jean-Simon Rech, Antony Canellas, Camille Rolland-Debord, Nadege Lemarié, Nicolas Belaube, Marine Nadal, Martin Siguier, Camille Petit-Hoang, Julie Chas

***Local Clinical Research unit:*** Elodie Drouet, Matthieu Lemoine, Audrey Phibel, Lucie Aunay, Eliane Bertrand, Sylviane Ravato, Marie Vayssettes, Anne Adda, Celine Wilpotte, Pélagie Thibaut

***Pharmacy:*** Julie Fillon, Isabelle Debrix

***Biological resource centre:*** Soraya Fellahi, Jean-Philippe Bastard, Guillaume Lefévre

**Hôpitaux Universitaires de Strasbourg, Université de Strasbourg**

***Co-PIs:*** Jacques-Eric Gottenberg and Yves Hansmann

***Investigators:*** Frédéric Blanc, Sophie Ohlmann-Caillard, Vincent Castelain, Emmanuel Chatelus, Eva Chatron, Olivier Collange, François Danion, Frédéric De Blay, Pierre Diemunsch, Sophie Diemunsch, Renaud Felten, Bernard Goichot, Valentin Greigert, Aurelien Guffroy, Bob Heger, Charlotte Kaeuffer, Loic Kassegne, Anne Sophie Korganow, Pierrick Le Borgne, Nicolas Lefebvre, Paul-Michel Mertes, Eric Noll, Mathieu Oberlin, Vincent Poindron, Julien Pottecher, Yvon Ruch, François Weill

***Local Clinical Research unit:*** Nicolas Meyer, Emmanuel Andres, Eric Demonsant, Hakim Tayebi, Gabriel Nisand, Stéphane Brin, Cédric Sublon

***Pharmacy:*** Guillaume Becker, Anne Hutt, Tristan Martin

***Biological Resource Centre:*** Sophie Bayer, Catherine Metzger

**AP-HP, Hôpital Saint-Antoine, Université Paris-Sorbonne**

***PI:*** Arsene Mekinian, ***Co-PI:*** Karine Lacombe, Bertrand Guidet

***Investigators:*** Noémie Abisror, Amir Adedjouma, Diane Bollens, Marion Bonneton, Nathalie Bourcicaux, Anne Bourrier, Maria Chauchard Thibault Chiarabiani, Doroth.e Chopin, Jonathan Cohen, Ines Devred, Bruno Donadille, Olivier Fain, Geoffrey Hariri, Vincent Jachiet, Patrick Ingliz, Marc Garnier, Marc Gatfosse, Etienne Ghrenassia, Delphine Gobert, Bertrand Guidet, Jessica Krause le Garrec, Cecilia Landman, Jean Remy Lavillegrand, Benedicte Lefebvre, Thibault Mahevas, Sandie Mazerand, Jean Luc Meynard, Marjolaine Morgand, Zineb Ouaz.ne, Jerome Pacanowski, S.bastien Riviere, Philippe Seksik, Harry Sokol, Heithem Soliman, Nadia Valin, Thomas Urbina

***Local Clinical Research unit:*** Chloé McAvoy, Maria Pereira Miranda, Gladys Aratus, Laurence Berard, Tabassome Simon,

***Pharmacy:*** Anne Daguenel Nguyen, Elise Girault, Cl.mentine Mayala-Kanda, Marie Antignac, Céline Leplay

***Biological resource centre:*** Gladys Aratus, Laurence Berard, Tabassome Simon

**AP-HP, Hôpital Européen Georges Pompidou, Université de Paris**

***PI:*** Jean-Benoit Arlet, ***Co-PI:*** Jean-Luc Diehl

***Investigators:*** Florence Bellenfant, Anne Blanchard, Alexandre Buffet, Bernard Cholley, Antoine Fayol, Edouard Flamarion, Anne Godier, Thomas Gorget, Sophie-Rym Hamada, Caroline Hauw-Berlemont, Jean-Sébastien Hulot, David Lebeaux, Marine Livrozet, Adrien Michon, Arthur Neuschwander, Marie-Aude Pennet, Benjamin Planquette, Brigitte Ranque, Olivier Sanchez, Geoffroy Volle

***Local Clinical Research unit:*** Sandrine Briois, Mathias Cornic, Virginie Elisee, Jesuthasan Denis, Juliette Djadi-Prat, Pauline Jouany, Ramon Junquera, Mickael Henriques, Amina Kebir, Isabelle Lehir, Jeanne Meunier, Florence Patin, Val.rie Paquet, Anne Tréhan, Véronique Vigna

***Pharmacy:*** Brigitte Sabatier

***Biological resource centre, Clinical Investigation Centre, PARCC, Inserm:*** Damien Bergerot, Charléne Jouve, Camille Knosp, Olivia Lenoir, Nassim Mahtal, Léa Resmini

**AP-HP, Hôpital Bichat, Université de Paris**

***PI:*** Xavier Lescure, ***Co-PI:*** Jade Ghosn

***Investigators:*** Antoine Bachelard, Anne Rachline, Valentina Isernia, Bao-chau, Phung, Dorothée Vallois, Aurelie Sautereau, Catherine Neukrich, Antoine Dossier, Raphaël Borie, Bruno Crestani, Gregory Ducrocq, Philippe Gabriel Steg, Philippe Dieude, Thomas Papo

***Local Clinical Research unit:*** Estelle Marcault, Marhaba Chaudhry, Charléne Da Silveira, Annabelle Metois, Ismahan Mahenni, Meriam Meziani, Cyndie Nilusmas

***Local Clinical Research team:*** Sylvie Le Gac, Awa Ndiaye, Fran.oise Louni, Malikhone Chansombat, Zelie Julia, Solaya Chalal, Lynda Chalal

***Pharmacy:*** Laura Kramer, Jeniffer Le Grand

***Biological resource centre:*** Kafif Ouifiya, Valentine Piquard, Sarah Tubiana

**AP-HP, Hôpital Beaujon, Université de Paris**

***PI:*** Yann Nguyen

***Investigators:*** Vasco Honsel, Emmanuel Weiss, Anais Codorniu, Virginie Zarrouk, Victoire de Lastours, Matthieu Uzzan

***Local Clinical Research unit:*** Naura Gamany, Agathe Claveirole, Alexandre Navid, Tiffanie Fouque, Yonathan Cohen, Maya Lupo, Constance Gilles, Roza Rahli

***Pharmacy:*** Zeina Louis

**AP-HP, Hôpital Saint-Louis, Université de Paris**

***PI:*** David Boutboul, ***Co-PI:*** Lionel Galicier, Elie Azoulay

***Investigators:*** Lionel Galicier, Yaël Amara, Gabrielle Archer, Elie Azoulay, Amira Benattia, Anne Bergeron, Louise Bondeelle, Nathalie de Castro, Melissa Clément, Michaël Darmon, Blandine Denis, Clairelyne Dupin, Elsa Feredj, Delphine Feyeux, Adrien Joseph, Etienne

Lenglin., Pierre Le Guen, Geoffroy Liégeon, Gwenaël Lorillon, Asma Mabrouki, Eric Mariotte, Grégoire Martin de Frémont, Adrien Mirouse, Jean-Michel Molina, Régis Peffault de Latour, Eric Oksenhendler, Julien Saussereau, Abdellatif Tazi, Jean-Jacques Tudesq, Lara Zafrani

***Local Clinical Research team:*** Isabelle Brindele, Emmanuelle Bugnet, Karine Celli Lebras, Julien Chabert, Lamia Djaghout, Catherine Fauvaux, Anne Lise Jegu, Ewa Kozakiewicz, Martine Meunier, Marie-Thérèse Tremorin

***Pharmacy:*** Claire Davoine, Isabelle Madelaine

***Biological resource centre:*** Sophie Caillat-Zucman, Constance Delaugerre, Florence Morin

**AP-HP, Hôpital Lariboisière, Université de Paris**

***PI:*** Damien Sène

**Investigators:** Ruxandra Burlacu, Benjamin Chousterman, Bruno Mégarbanne, Pascal Richette, Jean-Pierre Riveline, Aline Frazier

***Local Clinical Research unit:*** Eric Vicaut, Laure Berton, Tassadit Hadjam, Miguel Alejandro Vazquez-Ibarra, Clément Jourdaine, Olivia Tran, Véronique Jouis

***Pharmacy:*** Aude Jacob, Julie Smati, Stéphane Renaud

***Biological resource centre:*** Claire Pernin, Lydia Suarez

**AP-HP, Hôpital Avicenne, Université Paris-Nord Sorbonne**

***PI:*** Luca Semerano

***Investigators:*** Sébastien Abad, Ruben B.nainous, Nicolas Bonnet, Celine Comparon, Yves Cohen, Hugues Cordel, Robin Dhote, Nathalie Dournon, Boris Duchemann, Nathan Ebstein, Thomas Gille, Benedicte Giroux-Leprieur, Jeanne Goupil de Bouille, Hilario Nunes, Johanna Oziel, Dominique Roulot, Lucile Sese, ClaireTantet, Yurdagul Uzunhan

***Local Clinical Research Unit:*** Coralie Bloch-Queyrat, Vincent Levy, Fadhila Messani, Mohammed Rahaoui, Myléne Petit

***Pharmacy:*** Sabrina Brahmi, Vanessa Rathoin, Marthe Rigal

**AP-HP, Hôpital Cochin, Université de Paris**

***PI:*** Nathalie Costedoat-Chalumeau ***Co-PI:*** Liem Binh Luong, Frédéric Pene

***Investigators:*** Zakaria Ait Hamou, Sarah Benghanem, Philippe Blanche, Nicolas Carlier, Benjamin Chaigne, Remy Gauzit, Hassan Joumaa, Mathieu Jozwiak, Marie Lachétre, Hélène Lafoeste, Odie Launay, Paul Legendre, Jonathan Marey, Caroline Morbieu, Lola-Jade Palmieri, Tali-Anne Szwebel

***Local Clinical Research unit:*** Hendy Abdoul, Alexandra Bruneau, Audrey Beclin-Clabaux, Charly Larrieu, Pierre Montanari, Eric Dufour

***Local Clinical Research team:*** Ada Clarke, Catherine Le Bourlout, Nathalie Marin, Nathalie Menage, Samira Saleh-Mghir, Mamadou Salif Cisse, Kahina Cheref

***Pharmacy:*** Corinne Guerin, Jérémie Zerbit

**AP-HP, Hôpital Henri Mondor, Université Paris-Est Créteil**

***PI:*** Marc Michel

***Investigators:*** Sébastien Gallien, Etienne Crickx, Benjamin Le Vavasseur, Emmanuelle Kempf, Karim Jaffal, William Vindrios, Julie Oniszczuk, Marc Michel, Matthieu Mahevas, Constance Guillaud, Frédéric Schlemmer, Pascal Lim, Elena Fois, Giovanna Melica, Marie Matignon, Maud Jalabert, Jean-Daniel Lelièvre

***Local Clinical Research unit:*** David Schmitz, Marion Bourhis, Sylia Belazouz, Laetitia Languille, Caroline Boucle, Nelly Cita, Agnés Didier, Fahem Froura, Katia Ledudal, Thiziri Sadaoui

***Pharmacy:*** Alaki Thiemele, Delphine Le Febvre De Bailly, Muriel Carvhalo Verlinde

**AP-HP, Hôpital de la Pitié-Salpêtrière, Université Paris-Sorbonne**

***PI:*** Julien Mayaux, ***Co-PI:*** Patrice Cacoub

***Investigators:*** David Saadoun, Mathieu Vautier, Héléne Bugaut, Olivier Benveniste, Yves Allenbach, Gaëlle Leroux, Aude Rigolet, Perrine Guillaume-Jugnot, Fanny Domont, Anne Claire Desbois, Chlo**é** Comarmond, Nicolas Champtiaux, Segolene Toquet, Amine Ghembaza, Matheus Vieira, Georgina Maalouf, Goncalo Boleto, Yasmina Ferfar, Jean-Christophe Corvol, C.line Louapre, Sara Sambin, Louise-Laure Mariani, Carine Karachi

***Local Clinical Research unit:*** Florence Tubach, Candice Estellat, Linda Gimeno, Karine Martin, Aicha Bah, Vixra Keo, Sabrine Ouamri, Yasmine Messaoudi, Nessima Yelles, Pierre Faye

***Local Clinical Research team:*** Sebastien Cavelot, Cecile Larcheveque, Laurence Annonay, Jaouad Benhida, Aida Zahrate-Ghoul, Soumeya Hammal, Ridha Belilita

***Pharmacy:*** Fanny Charbonnier

**AP-HP, Hôpital Necker Enfants Malades, Université de Paris**

***PI:*** Claire Aguilar

***Investigators:*** Fanny Alby-Laurent, Carole Burger, Clara Campos-Vega, Nathalie Chavarot, Benjamin Fournier, Claire Rouzaud, Damien Vimpére

***Local Clinical Research unit:*** Caroline Elie, Prissile Bakouboula, Laure Choupeaux, Sophie Granville, Elodie Issorat

***Pharmacy:*** Christine Broissand

***Biological resource centre:*** Marie-Alexandra Alyanakian

**AP-HP, Hôpital Ambroise Paré, Université Paris-Saclay**

***PI:*** Guillaume Geri

***Local Clinical Research unit:*** Nawal Derridj, Naima Sguiouar, Hakim Meddah, Mourad Djadel

***Pharmacy:*** Héléne Chambrin-Lauvray

**AP-HP, Hôpital Paul Brousse, Université Paris-Saclay**

***PI:*** Jean-Charles Duclos-vallée, ***Co-PI:*** Faouzi Saliba

***Investigators:*** Sophie-Caroline Sacleux, Ilias Kounis

***Local Clinical Research unit:*** Sonia Tamazirt

***Pharmacy:*** Eric Rudant

**Institut Gustave Roussy, Université Paris-Saclay**

***PI:*** Jean-Marie Michot

***Investigators:*** Annabelle Stoclin, Emeline Colomba, Fanny Pommeret, Christophe Willekens

***Local Clinical Research unit:*** Rosa Da Silva, Valérie Dejean, Yasmina Mekid, Ines Ben-Mabrouk

***Pharmacy:*** Florence Netzer

***Biological resource centre:*** Caroline Pradon, Laurence Drouard, Valérie Camara-Clayette

**Hôpital Privé d’Antony**

***PI:*** Alexandre Morel,

***Investigators:*** Gilles Garcia, Abolfazl Mohebbi

***Local Clinical Research unit:*** Férial Berbour, Mélanie Dehais

***Pharmacy:*** Anne-Lise Pouliquen, Alison Klasen, Loren Soyez-Herkert

**Groupe Hospitalier Diaconesse Croix Saint-Simon**

***PI:*** Jonathan London

***Investigators:*** Jonathan London

***Local Clinical Research unit:*** Younes Keroumi

***Pharmacy:*** Emmanuelle Guillot

**Hôpital de Valenciennes**

***PI:*** Guillaume Grailles

***Investigators:*** Younes El amine, Fanny Defrancq

***Local Clinical Research unit:*** Hanane Fodil, Chaouki Bouras

***Pharmacy:*** Dominique Dautel

**Hôpital Delafontaine de Saint Denis**

***PI:*** Nicolas Gambier

***Pharmacy:*** Thierno Dieye

**Hôpital Marseille - Hôpital Saint Joseph**

***PI:*** Boris Bienvenu

***Investigators:*** Victor Lancon

***Local Clinical Research unit:*** Laurence Lecomte, Kristina Beziriganyan, Belkacem Asselate

***Pharmacy:*** Laure Allanic, Elena Kiouris, Marie-Héléne Legros, Christine Lemagner, Pascal Martel, Vincent Provitolo

**Hôpital Foch- Suresnes**

***PI:*** Félix Ackermann

***Local Clinical Research unit:*** Mathilde Le Marchand

***Pharmacy:*** Aurélie Chan Hew Wai, Dimitri Fremont

**CHU de Clermont-Ferrand - Gabriel Montpied**

***PI:*** Elisabeth Coupez

***Local Clinical Research unit:*** Mireille Adda, Frédéric Duée

***Pharmacy:*** Lise Bernard

**CH André Mignot-Versailles**

***PI:*** Antoine Gros

***Local Clinical Research Unit:*** Estelle Henry

***Pharmacy:*** Claire Courtin, Anne Pattyn

**CHU Dijon–Bourgone**

***PI:*** Pierre-Grégoire Guinot

***Local Clinical Research unit:*** Marc Bardou, Agnes Maurer

***Pharmacy:*** Julie Jambon, Amélie Cransac, Corinne Pernot

**Hôpital Robert Debré – Reims**

***PI:*** Bruno Mourvillier

***Local Clinical Research Unit:*** Eric Marquis

***Pharmacy:*** Philippe Benoit

**AP-HP – Hôpital Louis Mourier**

***PI:*** Damien Roux

***Local Clinical Research unit:*** Coralie Gernez

**Hôpital Claude Huriez - Lille**

***PI:*** Cécile Yelnik, ***Co-PI:*** Julien Poissy

***Local Clinical Research unit:*** Mandy Nizard

***Pharmacy:*** Fanette Denies

**Centre Hospitalier Robert Ballanger - Aulnay-sous-Bois**

***PI:*** Helene Gros

**GH Paris Saint Joseph**

***PI:*** Jean-Jacques Mourad

***Local Clinical Research unit:*** Emmanuelle Sacco

***Pharmacy:*** Sophie Renet

**B. The DisCoVeRy Study Group:**
**The French DisCoVeRy Trial Management Team:**
F Ader, Y Yazdanpanah, F Mentre, N Peiffer-Smadja, FX Lescure, J Poissy, L Bouadma, JF Timsit, B Lina, F Morfin-Sherpa, M Bouscambert, A Gaymard, G Peytavin, L Abel, J Guedj, C Andrejak, C Burdet, C Laouenan, D Belhadi, A Dupont, T Alfaiate, B Basli, A Chair, S Laribi, J Level, M Schneider, MC Tellier, A Dechanet, D Costagliola, B Terrier, M Ohana, S Couffin-Cadiergues, H Esperou, C Delmas, J Saillard, C Fougerou, L Moinot, L Wittkop, C Cagnot, S Le Mestre, D Lebrasseur-Longuet, V Petrov-Sanchez, A Diallo, N Mercier, V Icard, B Leveau, S Tubiana, B Hamze, A Gelley, M Noret, E D’Ortenzio, O Puechal, C Semaille.

**The DisCoVeRy Steering Committee** (members who are not listed in other groups)**:** T Welte, JA Paiva, M Halanova, MP Kieny
**ANRS (France Recherche Nord&Sud SIDA-HIV Hépatites), Paris, France:** E Balssa, C Birkle, S Gibowski, E Landry, A Le Goff, L Moachon, C Moins, L Wadouachi, C Paul, A Levier
**Centre Hospitalier Annecy Genevois, France:** D Bougon
**Centre Hospitalier de Cayenne Andrée Rosemon, Cayenne, France:** F Djossou, L Epelboin **Centre Hospitalier Universitaire de Nice, France:** J Dellamonica, CH Marquette
**Centre Hospitalier Régional de Metz-Thionville, France:** C Robert
**Centre Hospitalier Régional Universitaire de Nancy, France:** S Gibot
**Centre Hospitalier de Tourcoing, France:** E Senneville, V Jean-Michel
**Centre Hospitalier Universitaire de Amiens, France:** Y Zerbib
**Centre Hospitalier Universitaire de Besançon, France:** C Chirouze
**Centre Hospitalier Universitaire de Bordeaux, France:** A Boyer, C Cazanave, D Gruson, D Malvy
**Centre Hospitalier Universitaire de Dijon, France:** P Andreu, JP Quenot
**Centre Hospitalier Universitaire de Grenoble Alpes, France:** N Terzi
**Centre Hospitalier Universitaire de Lille, France:** K Faure
**Centre Hospitalier Universitaire de Martinique, Fort-de-France, France:** C Chabartier **Centre Hospitalier Universitaire de Montpellier, France:** V Le Moing, K Klouche
**Centre Hospitalier Universitaire de Lyon, France:** T Ferry, F, Valour
**Centre Hospitalier Universitaire de Nantes, France:** B Gaborit, E Canet, P Le Turnier, D Boutoille
**Centre Hospitalier Universitaire de Reims, France:** F Bani-Sadr  **Centre Hospitalier Universitaire de Rennes, France:** F Benezit, M Revest, C Cameli, A Caro, MJ Ngo Um Tegue, Y Le Tulzo, B Laviolle, F Laine
**Centre Hospitalier Universitaire de Saint-Étienne, France:** G Thiery
**Centre Hospitalier Universitaire de Strasbourg, France:** F Meziani, Y Hansmann, W Oulehri, C Tacquard **Centre Hospitalier Universitaire de Toulouse, France:** F Vardon-Bounes, B Riu-Poulenc, M Murris-Espin
**Centre Hospitalier Universitaire de Tours, France:** L Bernard, D Garot
**Groupe Hospitalier de Mulhouse Sud Alsace, France:** O Hinschberger **Hospices Civils de Colmar, France:** M Martinot
**Groupe Hospitalier de Paris Saint Joseph, Paris, France:** C Bruel, B Pilmis
**Hôpital Avicenne, Assistance Publique – Hôpitaux de Paris, France:** O Bouchaud
**Centre Hospitalier Universitaire de Nîmes, France:** P Loubet, C Roger
**Hôpital Bicêtre, Assistance Publique – Hôpitaux de Paris, France:** X Monnet, S Figueiredo **Hôpital Bichat - Claude Bernard, Assistance Publique – Hôpitaux de Paris, France:** V Godard
**Hôpital Cochin, Assistance Publique – Hôpitaux de Paris, France:** JP Mira, M Lachatre, S Kerneis
**Hôpital Delafontaine, Saint-Denis, France:** J Aboab, N Sayre, F Crockett
**Hôpital Européen Georges-Pompidou, Assistance Publique – Hôpitaux de Paris, France:** D Lebeaux, A Buffet, JL Diehl, A Fayol, JS Hulot, M Livrozet
**Hôpital Henri-Mondor, Assistance Publique – Hôpitaux de Paris, France:** A Mekontso- Dessap
**Hôpital d'Instruction des Armées Bégin, Saint Mandé, France:** C Ficko
**Hôpital Marie Lannelongue, Le Plessis Robinson, France:** F Stefan, J Le Pavec
**Hôpital de la Pitié-Salpêtrière, Assistance Publique – Hôpitaux de Paris, France:** J Mayaux **Hôpital Saint-Antoine, Assistance Publique – Hôpitaux de Paris, France:** H Ait-Oufella **Hôpital Saint-Louis, Assistance Publique – Hôpitaux de Paris, France:** JM Molina

**Hôpital Tenon, Assistance Publique – Hôpitaux de Paris, France:** G Pialoux, M Fartoukh **Hospices Civils de Lyon, France:** J Textoris
**Inserm U1018, Université Paris Saclay, CESP, Paris, France:** M Brossard, A Essat **Inserm US19-Sc10, Université Paris Saclay, Villejuif, France:** E Netzer, Y Riault, M Ghislain **Sorbonne Université, INSERM U1136, Institut Pierre Louis d'Épidémiologie et de Santé Publique (IPLESP), Paris, France:** L Beniguel, M Genin, L Gouichiche
**CMG Inserm U1219, Bordeaux Population Health, Université de Bordeaux, Bordeaux, France:** L Moinot, C Betard, L Wittkop **Cliniques Universitaires de Saint Luc, Bruxelles, Belgique:** L Belkhir
**Centre Hospitalier Régional de la Citadelle, Liège, Belgique:** A Altdorfer, V Fraipont Centro **Hospital Universitário de Lisboa Norte, Hospital de Santa Maria, Portugal:** S Braz, JM Ferreira Ribeiro
**Centro Hospitalar Universitário São João de Porto, Portugal:** JA Paiva, R Roncon Alburqueque
**Hôpitaux Robert Schuman, Luxembourg:** M Berna
**Luxembourg Institute of Health, Strassen, Luxembourg:** M Alexandre
**Kepler Universitätsklinikum Linz, Linz, Austria:** B Lamprecht
**Paracelsus Medical University Salzburg, SCRI-CCCIT and AGMT, Austria:** A Egle, R Greil
**AGMT Arbeitsgemeinschaft Medikamentöse Tumortherapie, Salzburg, Austria:** R Greil **Medizinische Universität Innsbruck, Innsbruck, Austria:** M Joannidis

**C. ACTT-2 Study Group**

(listed in PubMed, and ordered by enrollment)

The following study group members were all closely involved with the design, implementation,

and oversight of the ACTT-2 study.

**University of Texas Health San Antonio, University Health, and the South Texas Veterans Health Care System, San Antonio, TX.** Thomas F. Patterson, M.D., Philip O. Ponce, M.D., Barbara S. Taylor, M.D., M.S., Jan E. Patterson, M.D., M.S., Jason E. Bowling, M.D., Heta Javeri, M.D.

**University of Nebraska Medical Center, Omaha, NE.** Andre C. Kalil, M.D., M.P.H., LuAnn

Larson, BSN, Angela Hewlett, M.D., M.S.

**Emory University, Atlanta, GA.** Aneesh K. Mehta, M.D., Nadine G. Rouphael, M.D., Youssef

Saklawi, M.D., Nicholas Scanlon, M.D., Jessica J. Traenkner, P.A.-C, Ronald P. Trible Jr., M.D.,

Ph.D.

**Duke University, Durham, NC.** Emmanuel B. Walter, M.D., M.P.H., Noel Ivey, M.D., Thomas L. Holland, M.D., M.Sc

**Instituto Nacional de Ciencias Médicas y Nutrición Salvador Zubiran, Mexico City, Mexico.** Guillermo M. Ruiz-Palacios, M.D., Alfredo Ponce de León, M.D., Sandra Rajme, M.D.

**University of California Irvine, Irvine, CA.** Lanny Hsieh, M.D., Alpesh N. Amin, M.D., Miki

Watanabe, MD, Helen S. Lee, Pharm.D., BCIDP.

**University of Minnesota, Minneapolis, MN.** Susan Kline, M.D., M.P.H., Joanne Billings, M.D., M.P.H., Brooke Noren, R.N., Hyun Kim, MD, Tyler D. Bold, MD, PhD.

**Cedars Sinai Medical Center, Los Angeles, CA.** Victor Tapson, M.D., Jonathan Grein, M.D.,

Fayyaz Sutterwala, M.D.

**University of Florida Health-Shands Hospital, Gainesville, FL.** Dr. Nicole Iovine, M.D., Lars K. Beattie, M.D., Rebecca Murray Wakeman, M.D., and Matthew Shaw, M.D.

**UT Southwestern Medical Center, & Parkland Health & Hospital System, Dallas, TX.** Mamta K. Jain, M.D., M.P.H., Satish Mocherla, M.D., Jessica Meisner, M.D., Amneris Luque, M.D.

**University of California San Diego, La Jolla, CA.** Daniel A. Sweeney, M.D., Constance A.

Benson, M.D., Farhana Ali, M.D.

**Baylor College of Medicine, Houston, TX.** Robert L. Atmar, M.D., Hana M. El Sahly, M.D.,

Jennifer Whitaker, M.D.

**University of Rochester Medical Center, Rochester, NY.** Ann R. Falsey, M.D., Angela R.

Branche, M.D., Cheryl Rozario, M.D.

**Instituto Nacional de Enfermedades Respiratorias, Mexico City, Mexico.** Justino Regalado

Pineda, M.D. M.Sc., José Arturo Martinez-Orozco M.D.

**National Centre for Infectious Diseases/Tan Tock Seng Hospital, Singapore.** David Chien Lye, MBBS, Sean WX Ong MBBS, Po Ying Chia, MBBS, Barnaby E Young, MBBS

**Baylor Scott & White Health, Dallas, TX.** Uriel Sandkovsky, M.D., M.S, Mezgebe Berhe, M.D., M.P.H., Clinton Haley, M.D., M.P.H., Emma Dishner, M.D., M.P.H.

**Grady Memorial Hospital, Atlanta, GA.** Valeria D Cantos, M.D., Colleen F. Kelley, M.D.,

M.P.H., Paulina A. Rebolledo Esteinou M.D., M.Sc., Sheetal Kandiah, M.D., M.P.H.

**University of California San Francisco, San Francisco, CA.** Sarah B. Doernberg, M.D., Pierre-Cedric B. Crouch, Ph.D., R.N., ANP-BC, ACRN, Hannah Jang, Ph.D., R.N., CNL, PHN

Zuckerberg San Francisco General, San Francisco, CA. Anne F. Luetkemeyer, M.D., Jay Dwyer,

RN.

**University of California Davis, Davis, CA.** Stuart H. Cohen M.D., George R. Thompson 3rd,

M.D., Hien H. Nguyen, M.D., M.A.S.

**University of Massachusetts Medical School, Worcester, MA.** Robert W. Finberg, M.D., Jennifer P. Wang, M.D., Juan Perez-Velazquez, MD, Mireya Wessolossky, M.D.

**University of Virginia, Charlottesville, VA.** Patrick E. H. Jackson M.D., Taison D. Bell, M.D.,

Miranda J. West, M.S.

**Northwestern University, Chicago, IL.** Babafemi Taiwo, M.B.B.S., Karen Krueger M.D., Johnny Perez, RN, BSN., Triniece Pearson, PhD, MBA, RN, CCRC

**Pennsylvania State Health Milton S. Hershey Medical Center, Hershey, PA.** Catharine I. Paules, M.D., Kathleen G. Julian MD, Danish Ahmad MD, Alexander G. Hajduczok MD

**Providence Sacred Heart Medical Center, Spokane, WA.** Henry Arguinchona M.D.,

Christa Arguinchona MSN, R.N., CCRN.

**University of Alabama at Birmingham (UAB), Birmingham, AL.** Nathaniel Erdmann M.D.,

Ph.D., Paul Goepfert M.D.

**Stanford University, Stanford, CA.** Neera Ahuja, M.D.

**Denver Health and Hospital Authority, Denver, CO.** Maria G. Frank, M.D, David Wyles, M.D., Heather Young, M.D.

**Seoul National University Hospital, Seoul, Korea.** Myoung-don Oh, M.D., Wan Beom Park,

M.D., Chang Kyung Kang, M.D.

**Atlanta VA Medical Center, Atlanta, GA.** Vincent Marconi, M.D., Abeer Moanna, M.D.,

Sushma Cribbs M.D., Telisha Harrison, M.S.W.

**Seoul National University Bundang Hospital, Seongnam, Korea.** Eu Suk Kim, M.D., Jongtak

Jung, M.D., Kyoung-Ho Song, M.D., Hong Bin Kim, M.D.

**Changi General Hospital, Singapore.** Seow Yen Tan, MBBS, Humaira Shafi, MBBS, Jaime MF Chien, MBBS, Raymond KC Fong, MBBS

**Rigshospitalet, Dept. of Infectious Diseases (CHIP), Copenhagen, Denmark.** Daniel D. Murray, Ph.D., Jens Lundgren, M.D.

**Aalborg University Hospital, Dept. of Infectious Diseases, Denmark.** Henrik Nielsen, M.D.

**Nord Zealand University Hospital, Dept. of Infectious Diseases, Denmark.** Tomas Jensen, M.D.

**Montefiore Medical Center/Albert Einstein College of Medicine, Bronx, NY.** Barry S. Zingman, M.D., Robert Grossberg, M.D., Paul F. Riska, M.D.

**University of California Los Angeles, Los Angeles, CA.** Otto O. Yang M.D., Jenny Ahn R.N.

B.S.N., Rubi Arias B.A.

**University of Maryland School of Medicine, Baltimore, MD.** Rekha R. Rapaka, M.D. Ph.D.,

Naomi Hauser, M.D., James D. Campbell, M.D., M.S.

**University of Pennsylvania, Philadelphia, PA.** William R. Short, M.D. M.P.H., Pablo Tebas, M.D., Jillian T. Baron, M.D., M.P.H.

**University of Texas Medical Branch, Galveston, TX.** Susan L. F. McLellan MD, MPH, Lucas S. Blanton, MD, Justin B. Seashore MD

**Vanderbilt University Medical Center, Nashville, TN.** C. Buddy Creech, M.D., M.P.H., Todd W. Rice, M.D., MSCI; Shannon Walker, MD; Isaac P. Thomsen, MD, MSCI

**Evergreen Health Medical Center, Kirkland, WA.** Diego Lopez de Castilla, M.D. M.P.H., Jason W. Van Winkle M.D., Francis X. Riedo M.D.

**Ng Teng Fong General Hospital, Singapore.** Surinder Kaur Pada, M.D., MBBS, MPH, Alvin DY Wang, MBBS, Li Lin, MD

**University of New Mexico Health Sciences Center, Albuquerque, NM.** Michelle Harkins, MD, Gregory Mertz MD, Nestor Sosa MD

**National University Hospital, Singapore.** Louis Yi Ann Chai, MBBS, PhD, Paul Anantharajah

Tambyah, MBBS, Sai Meng Tham, MBBS, Sophia Archuleta, M.D., Gabriel Yan, MBBS

**Brooke Army Medical Center; and IDCRP, USUHS, San Antonio, TX.** David A. Lindholm,

M.D., Ana Elizabeth Markelz, M. D.

**IDCRP, USUHS; HJF; and Brooke Army Medical Center, San Antonio, TX.** Katrin Mende,

Ph.D.

**Kaiser Permanente Northwest, Portland, OR.** Richard Mularski, M.D., M.S.H.S, M.C.R.

**Massachusetts General Hospital, Boston, MA.** Elizabeth Hohmann, M.D., Mariam Torres-Soto MA and Nikolaus Jilg M.D. Ph.D.

**Naval Medical Center, San Diego; and IDCRP, USUHS, San Diego, CA.** Ryan C. Maves, M.D.

**IDCRP, USUHS; HJF; and Naval Medical Center San Diego, San Diego, CA.** Gregory C. Utz, M.D.

**Saint Louis University, Saint Louis, MO.** Sarah L. George, M.D., Daniel F. Hoft, M.D., Ph.D.,

James D. Brien, Ph.D.

**Hospital Germans Trias i Pujol, Badalona, Spain.** Roger Paredes, M.D., Ph.D., Lourdes Mateu, M.D., Ph.D., Cora Loste, M.D.

**Georgetown University, Washington, DC.** Princy Kumar, M.D., Sarah Thornton, MD

Sharmila Mohanraj, MD

**Johns Hopkins University School of Medicine, Baltimore, MD.** Noreen A. Hynes, M.D., M.P.H., Lauren M. Sauer, M.S.

**Madigan Army Medical Center; and IDCRP, USUHS, Tacoma, WA.** Christopher J. Colombo, M.D., M.A., Christina Schofield, M.D.

**IDCRP, USUHS; HJF; and Madigan Army Medical Center, Tacoma, WA.** Rhonda E. Colombo, M.D., M.H.S.

**IDCRP, USUHS, Bethesda, MD; HJF, Bethesda, MD; and Madigan Army Medical Center,**

**Tacoma, WA.** Susan E Chambers, B.S.N., R.N.

**University of Illinois at Chicago, Chicago, IL.** Richard M. Novak, M.D., Andrea Wendrow RPh

**Indiana University, Indianapolis, IN.** Samir K. Gupta, M.D.

**Naval Medical Center Portsmouth; and IDCRP, USUHS, Portsmouth, VA.** Tida Lee, M.D.,

Ph.D.

**IDCRP, USUHS; HJF; and Naval Medical Center Portsmouth, Portsmouth, VA.** Tahaniyat

Lalani, M.B.B.S., M.H.S.

**VA Palo Alto Health Care System, Palo Alto, CA.** Mark Holodniy, M.D., Aarthi Chary, M.D.

**Walter Reed National Military Medical Center; and Infectious Disease Clinical Research**

**Program (IDCRP), Uniformed Services University of the Health Sciences (USUHS), Bethesda, MD.** Nikhil Huprikar, M.D.

**IDCRP, USUHS; Henry M. Jackson Foundation for the Advancement of Military Medicine, Inc. (HJF); and Walter Reed National Military Medical Center, Bethesda, MD.** Anuradha Ganesan, M.B.B.S., M.P.H

**National Center for Global Health and Medicine Hospital, Tokyo, Japan.** Norio Ohmagari, M.D., Ph.D, Ayako Mikami, M.D., Ph.D.

**Royal Victoria Infirmary, Newcastle, United Kingdom.** D. Ashley Price, MRCP; Christopher J. A. Duncan, MRCP.

**NYU Langone Health and NYC Health + Hospitals/ Bellevue, New York City, NY.** Kerry

Dierberg, M.D. M.P.H., Henry J Neumann, M.D.

**Ochsner Medical Center Kenner, Kenner, LA.** Stephanie N. Taylor, M.D., Alisha Lacour, M.D., Najy Masri, M.D.

**Southeast Louisiana Veterans Health Care System, New Orleans, LA.** Edwin Swiatlo, M.D.,

Kyle Widmer, M.D.

**University of Minnesota, School of Public Health and INSIGHT, Minneapolis, MN.** James D. Neaton, Ph.D.

**Veterans Affairs Eastern Colorado Healthcare System, Aurora, CO.** Mary Bessesen, M.D.

**Infectious Diseases Clinical Research Consortium (IDCRC), Emory University, Atlanta, GA.** David S. Stephens, M.D.

**Infectious Disease Clinical Research Program, Uniformed Services University of the Health**

**Sciences, Bethesda, MD.** Timothy H. Burgess, M.D. M.P.H.

**US Centers for Disease Control and Prevention Atlanta, GA.** Timothy M Uyeki, M.D. M.P.H

**Biomedical Advanced Research and Development Authority, Washington, DC.** Robert Walker, M.D., G. Lynn Marks, M.D

**Gilead Sciences, Foster City, CA.** Anu Osinusi, M.D. M.P.H., Huyen Cao, M.D.

**Eli Lilly, Indianapolis, IN.** Anabela Cardoso, M.D., M.B.A., Stephanie de Bono, M.D. Ph.D.,

Douglas E Schlichting

**Uniformed Services University of the Health Sciences, Bethesda, MD.** Kevin K. Chung, M.D.

**The Emmes Company, LLC, Rockville, MD.** Jennifer L. Ferreira, ScM; Michelle Green, MPH, MBA; Mat Makowski, PhD; Michael R.Wierzbicki, PhD; Tom M. Conrad, PhD; Jill Ann El-Khorazaty, MS; Heather Hill M.S.

**Clinical Monitoring Research Program Directorate, Frederick National Laboratory for Cancer Research, Frederick, MD.** Tyler Bonnett, M.S., Nikki Gettinger M.P.H, Theresa Engel, M.F.S., Teri Lewis, B.S., Jing Wang M.S.

**National Institute of Allergy and Infectious Diseases, National Institutes of Health, Bethesda, M.D.** John H. Beigel, M.D., Kay M. Tomashek, M.D., M.P.H., Varduhi Ghazaryan, MD.**,** MPH., Tatiana Beresnev, M.D. Seema Nayak, M.D., Lori E. Dodd, Ph.D., Walla Dempsey,

Ph.D., Effie Nomicos R.N., M.S.N., Marina Lee, Ph.D., , Rhonda Pikaart-Tautges, Mohamed

Elsafy M.D., Robert Jurao, R.N, B.S.N., Hyung Koo, R.N., BSN, Michael Proschan, Ph.D.,

Tammy Yokum, M.S.N., R.N., Janice Arega, M.S., Ruth Florese, MPH, PhD., Jocelyn D. Voell,

R.N., M.S., Richard Davey, M.D.

1. **ACTT-3 Study Group**

(listed in PubMed, and ordered by enrollment)

The following study group members were all closely involved with the design, implementation, and oversight of the ACTT-3 study.

**University of Nebraska Medical Center, Omaha, NE.** Andre C. Kalil, M.D., M.P.H., LuAnn Larson, BSN, Angela Hewlett, M.D., M.S.

**University of Texas Health San Antonio, University Health, and the South Texas Veterans Health Care System, San Antonio, TX.** Thomas F. Patterson, M.D., Philip O. Ponce, M.D., Jan E. Patterson, M.D., M.S., Barbara S. Taylor, M.D., M.S., Jason E. Bowling, M.D., Ruth C. Serrano, M.D.

**Emory University, Atlanta, GA.** Aneesh K. Mehta, M.D., Jessica J. Traenkner, P.A.-C, Nadine G. Rouphael, M.D., Zanthia Wiley, M.D., Varun K. Phadke, M.D.

**University of Alabama at Birmingham (UAB), Birmingham, AL.** Nathaniel Erdmann M.D., Ph.D., Paul A. Goepfert M.D.

**University of Utah, Salt Lake City, UT.** Carlos A. Gomez, M.D., Theresa A. Sofarelli, PA-C, Laura Certain, M.D., Hannah N. Imlay, M.D.

**Duke University, Durham, NC.** Cameron R. Wolfe, M.B.B.S., M.P.H., Emily R. Ko, M.D., Ph.D., John J. Engemann, M.D., Emmanuel B. Walter, M.D., M.P.H.

**UT Southwestern Medical Center Parkland Health & Hospital System, Dallas, TX.** Mamta K. Jain, M.D., M.P.H., Satish Mocherla, M.D., Jessica Meisner, M.D.

**Instituto Nacional de Ciencias Médicas y Nutrición Salvador Zubiran, Mexico City, Mexico.** Guillermo M. Ruiz-Palacios, M.D., Alfredo Ponce de León, M.D., Sandra Rajme, M.D.

**University of Minnesota Medical School, Minneapolis, MN.** Susan Kline, M.D., M.P.H., Joanne Billings, M.D., M.P.H., Hyun Kim, M.D.

**Instituto Nacional de Enfermedades Respiratorias, Mexico City, Mexico.** Justino Regalado Pineda, M.D. M.Sc., José Arturo Martinez-Orozco M.D., Nora Bautista Felix Ph.D.

**Emory Decatur, Decatur, GA.** Claire R. Wan, M.P.H, P.A.-C, Sammy T. Elmor, B.S., Laurel R. Bristow, M.Sc.

**University of New Mexico Health Sciences Center, Albuquerque, NM.** Michelle S. Harkins MD, Gregory Mertz MD, Nestor Sosa MD

**University of Virginia, Charlottesville, VA.** Patrick E. H. Jackson, M.D., Taison D. Bell, M.D., Miranda J. West, M.S.

**University of Florida Health-Shands Hospital, Gainesville, FL.** Nicole M. Iovine, M.D., Ph.D., Marie-Carmelle Elie-Turenne, M.D.

**Cedars Sinai Medical Center, Los Angeles, CA.** Victor F. Tapson, M.D., Jonathan Grein, M.D., Fayyaz Sutterwala, M.D.

**Seoul National University Hospital, Seoul, Korea.** Myoung-don Oh, M.D., Pyoeng Gyun Choe, M.D., M.P.H., Chang Kyung Kang, M.D.

**Kaiser Permanente Northwest, Portland, OR.** Richard A. Mularski, M.D., M.S.H.S, M.C.R.

**Pennsylvania State Health Milton S. Hershey Medical Center, Hershey, PA.** Catharine I. Paules, M.D., Kevin S. Rhie, M.D., Rezhan H. Hussein, M.D.

**Carver College of Medicine, University of Iowa, Iowa City, Iowa.** Dilek Ince, M.D., Patricia L. Winokur, M.D.

**National Center for Global Health and Medicine Hospital, Tokyo, Japan.** Jin Takasaki, M.D., Ayako Mikami, M.D., Ph.D., Sho Saito, M.D.

**University of California San Diego, La Jolla, CA.** Daniel A. Sweeney, M.D., Constance A. Benson, M.D., Kimberly McConnell, PharmD

**Baylor Scott & White Health, Dallas, TX.** Uriel Sandkovsky, M.D., M.S, Mezgebe Berhe, M.D., M.P.H., Emma Dishner, M.D., M.P.H.

**Denver Health and Hospital Authority, Denver, CO.** David L. Wyles, M.D., Maria G. Frank, M.D, Ellen Sarcone, M.D.

**UT Southwestern Medical Center, Dallas, TX.** Mamta K. Jain, M.D., M.P.H., Satish Mocherla, M.D., Jessica Meisner, M.D.

**University of California San Francisco, San Francisco, CA.** Sarah B. Doernberg, M.D., M.A.S., Pierre-Cedric B. Crouch, Ph.D., R.N., ANP-BC, ACRN, Hannah Jang, Ph.D., R.N., CNL, PHN

**Massachusetts General Hospital, Boston, MA.** Elizabeth Hohmann, M.D., Nikolaus Jilg, M.D., Ph.D.

**Houston Methodist, Houston, TX.** Kevin A. Grimes, M.D., M.P.H., Katherine Perez, PharmD, BCIDP, Charles Janak, PharmD, BCPS

**Ben Taub Hospital, Houston, TX.** Robert L. Atmar, M.D., Hana M. El Sahly, M.D., Jennifer A. Whitaker, M.D.

**Grady Memorial Hospital, Atlanta, GA.** Valeria D Cantos, M.D., Paulina A. Rebolledo M.D., M.Sc., John Gharbin, MBChB, M.P.H.

**Montefiore Medical Center/Albert Einstein College of Medicine, Bronx, NY.** Robert Grossberg, M.D., Barry S. Zingman, M.D., Paul F. Riska, M.D

**Providence Sacred Heart Medical Center, Spokane, WA.** Allison A. Lambert, M.D., M.H.S.,

Henry Arguinchona M.D., Christa Arguinchona MSN, R.N., CCRN.

**Evergreen Health Medical Center, Kirkland, WA.** Diego Lopez de Castilla, M.D. M.P.H., Jason W. Van Winkle M.D., Diego F. Zea M.D.

**Seoul National University Bundang Hospital, Seongnam, Korea.** Eu Suk Kim, M.D., Jongtak Jung, M.D., Kyoung-Ho Song, M.D., Hong Bin Kim, M.D.

**Zuckerberg San Francisco General Hospital, San Francisco, CA**. Anne F. Luetkemeyer, M.D., Jay Dwyer, R.N., Emma Bainbridge, M.D.

**Womack Army Medical Center, Ft. Bragg, NC.** David C. Hostler, M.D., M.P.H.; Jordanna M. Hostler, MD; Brian T. Shahan, M.D.

**University of California Irvine, Irvine, CA.** Lanny Hsieh, M.D., Alpesh N. Amin, M.D., Miki Watanabe, MD.

**University of Pennsylvania, Philadelphia, PA.** William R. Short, M.D. M.P.H., Pablo Tebas, M.D., Jillian T. Baron, M.D., M.P.H.

**Stanford University, Stanford, CA.** Neera Ahuja, M.D., Evelyn Ling, M.D., Minjoung Go, M.D.

**University of California Los Angeles, Los Angeles, CA.** Otto O. Yang M.D., Jenny Ahn R.N. B.S.N., Rubi Arias B.A.

**University of Maryland School of Medicine, Baltimore, MD.** Rekha R. Rapaka, M.D., Ph.D., Fleesie A. Hubbard, M.S., James D. Campbell, M.D., M.S.

**University of California Davis, Davis, CA.** Stuart H. Cohen M.D., George R. Thompson 3rd, M.D., Melony Chakrabarty, M.D.

**Rochester General Hospital, Rochester, NY.** Maryrose Laguio-Vila, M.D., Edward E. Walsh, M.D., Ann R. Falsey, M.D.

**Ochsner Medical Center Kenner, Kenner, LA.** Stephanie N. Taylor, M.D., Najy Masri, M.D., Alisha Lacour, M.D.

**Naval Medical Center Portsmouth; and IDCRP, USUHS, Portsmouth, VA.** Tida Lee, M.D., Ph.D.

**IDCRP, USUHS; HJF; and Naval Medical Center Portsmouth, Portsmouth, VA.** Tahaniyat Lalani, M.B.B.S., M.H.S.

**Baylor College of Medicine, Houston, TX.** Robert L. Atmar, M.D., Hana M. El Sahly, M.D., Jennifer A. Whitaker, M.D.

**Brooke Army Medical Center; and IDCRP, USUHS, San Antonio, TX.** David A. Lindholm, M.D., Ana Elizabeth Markelz, M.D

**IDCRP, USUHS; HJF; and Brooke Army Medical Center, San Antonio, TX.** Katrin Mende, Ph.D.

**University of Rochester Medical Center, Rochester, NY.** Ann R. Falsey, M.D., Angela R. Branche, M.D.

**Madigan Army Medical Center; and IDCRP, USUHS, Tacoma, WA.** Christopher J. Colombo, M.D., M.A., Christina Schofield, M.D.

**IDCRP, USUHS; HJF; and Madigan Army Medical Center, Tacoma, WA.** Rhonda E. Colombo, M.D., M.H.S.

**University of Florida College of Medicine, Jacksonville, FL.** Faheem Guirgis, M.D.

**VA Palo Alto Health Care System, Palo Alto, CA.** Mark Holodniy, M.D., Aarthi Chary, M.D.

**Veterans Affairs Eastern Colorado Healthcare System, Aurora, CO.** Mary Bessesen, M.D.

**Johns Hopkins University School of Medicine, Baltimore, MD.** Noreen A. Hynes, M.D., M.P.H., Lauren M. Sauer, M.S.

**Atlanta VA Medical Center, Atlanta, GA.** Vincent C. Marconi, M.D., Abeer Moanna, M.D., Telisha Harrison, M.S.W.

**National Centre for Infectious Diseases; Tan Tock Seng Hospital; Yong Loo Lin School of Medicine; Lee Kong Chian School of Medicine; Singapore.** David Chien Lye, M.B.B.S., Sean WX Ong M.B.B.S., Po Ying Chia, M.B.B.S.

**Walter Reed National Military Medical Center; and Infectious Disease Clinical Research Program (IDCRP), Uniformed Services University of the Health Sciences (USUHS), Bethesda, MD.** Nikhil Huprikar, M.D.

**IDCRP, USUHS; Henry M. Jackson Foundation for the Advancement of Military Medicine, Inc. (HJF); and Walter Reed National Military Medical Center, Bethesda, MD.** Anuradha Ganesan, M.B.B.S., M.P.H.

**Tripler Army Medical Center, Honolulu, HI.** Christian Madar, M.D.

**University of Illinois at Chicago, Chicago, IL.** Richard M. Novak, M.D., Andrea Wendrow RPh, Scott A. Borgetti, M.D.

**Saint Louis University, Saint Louis, MO.** Sarah L. George, M.D., Daniel F. Hoft, M.D., Ph.D., James D. Brien, Ph.D.

**University of Texas Medical Branch, Galveston, TX.** Susan L. F. McLellan M.D., M.P.H., Corri Levine M.S., Joy Nock, R.N., B.S.N.

**Changi General Hospital, Singapore.** Seow Yen Tan, MBBS, Humaira Shafi, MBBS, Jaime MF Chien, MBBS

**University of Miami, Miami, FL.** Keith Candiotti, M.D.

**University of Massachusetts Medical School, Worcester, MA.** Robert W. Finberg, M.D., Jennifer P. Wang, M.D., Mireya Wessolossky, M.D.

**Naval Medical Center, San Diego; and IDCRP, USUHS, San Diego, CA.** Ryan C. Maves, M.D.

**IDCRP, USUHS; HJF; and Naval Medical Center San Diego, San Diego, CA.** Gregory C. Utz, M.D.

**IDCRP, USUHS, Bethesda, MD; HJF, Bethesda, MD; and Madigan Army Medical Center, Tacoma WA.** Susan E Chambers, B.S.N., R.N.

**Infectious Diseases Clinical Research Consortium (IDCRC).** Robert L. Atmar, M.D.; David S. Stephens, M.D.

**Infectious Disease Clinical Research Program (IDCRP), Uniformed Services University of the Health Sciences, Bethesda, MD.** Timothy H. Burgess, M.D., M.P.H.

**Infectious Disease Clinical Research Program (IDCRP), Uniformed Services University of the Health Sciences, Bethesda, MD; Henry M. Jackson Foundation for the Advancement of Military Medicine, Inc.** Julia Rozman, B.S.

**EMD Serono Research & Development Institute, Inc., Billerica, MA, USA, an affiliate of Merck KGaA, Darmstadt, Germany.** Fernando Dangond, M.D.

**Merck KGaA, Darmstadt, Germany.** Yann Hyvert, Ph.D., Andrea Seitzinger, Ph.D.

**Gilead Sciences, Foster City, CA.** Anu Osinusi, M.D., M.P.H., Huyen Cao, M.D.

**Uniformed Services University of the Health Sciences, Bethesda, MD.** Kevin K. Chung, M.D.

**The Emmes Company, LLC, Rockville, MD.** Jennifer L. Ferreira, Sc.M.; Michelle Green, M.P.H., M.B.A; Mat Makowski, Ph.D.; Tom M. Conrad, Ph.D.; Kaitlyn Cross, M.S.; Jill Ann El-Khorazaty, M.S.; Heather Hill M.S., Stephanie Pettibone; Michael R. Wierzbicki, Ph.D.

**Clinical Monitoring Research Program Directorate, Frederick National Laboratory for Cancer Research, Frederick, MD.** Tyler Bonnett, M.S., Nikki Gettinger M.P.H, Theresa Engel, M.F.S., Teri Lewis, B.S., Jing Wang M.S.

**National Institute of Allergy and Infectious Diseases, National Institutes of Health, Bethesda, M.D.** John H. Beigel, M.D., Kay M. Tomashek, M.D., M.P.H., Varduhi Ghazaryan, M.D., M.P.H., Tatiana Beresnev, M.D., Seema U. Nayak, M.D., Lori E. Dodd, Ph.D., Walla Dempsey, Ph.D., Gregory A. Deye, M.D., Effie Nomicos R.N., M.S.N., Rhonda Pikaart-Tautges, Mohamed Elsafy M.D., Robert Jurao, R.N., B.S.N., Hyung Koo, R.N., B.S.N., Michael Proschan, Ph.D., Richard Davey, M.D., Tammy Yokum, M.S.N., R.N., Janice Arega, M.S., Ruth Florese, M.P.H., Ph.D.
